# Dependence of fluorescent protein brightness on protein concentration in solution and enhancement of it

**DOI:** 10.1038/srep22342

**Published:** 2016-03-09

**Authors:** Takamitsu J. Morikawa, Hideaki Fujita, Akira Kitamura, Takashi Horio, Johtaro Yamamoto, Masataka Kinjo, Akira Sasaki, Hiroaki Machiyama, Keiko Yoshizawa, Taro Ichimura, Katsumi Imada, Takeharu Nagai, Tomonobu M. Watanabe

**Affiliations:** 1 Graduate School of Frontier Biosciences, Osaka University, 1-3 Yamadaoka, Suita,Osaka 565-0871, JAPAN; 2 WPI, Immunology Frontier Research Center, Osaka University, 1-3 Yamadaoka, Suita, Osaka 565-0871, JAPAN; 3 The Institute of Scientific and Industrial Research, Osaka University, Ibaraki, Osaka 565-0873, JAPAN; 4 RIKEN Quantitative Biology Center (QBiC), Suita, Osaka 565-0874, JAPAN; 5 Faculty of Advanced Life Science, Hokkaido University, Sapporo 001-0021, JAPAN; 6 Biomedical Research Institute, National Institute of Advanced Industrial Science and Technology (AIST), Tsukuba, Ibaraki 305-8566, JAPAN; 7 Department of Macromolecular Science, Graduate School of Science, Osaka University, Toyonaka, Osaka 565-0043, JAPAN; 8 PRESTO, Japan Science and Technology Agency, 4-1-8 Honcho Kawaguchi, Saitama 332-0012, JAPAN

## Abstract

Fluorescent proteins have been widely used in biology because of their compatibility and varied applications in living specimens. Fluorescent proteins are often undesirably sensitive to intracellular conditions such as pH and ion concentration, generating considerable issues at times. However, harnessing these intrinsic sensitivities can help develop functional probes. In this study, we found that the fluorescence of yellow fluorescent protein (YFP) depends on the protein concentration in the solution and that this dependence can be enhanced by adding a glycine residue in to the YFP; we applied this finding to construct an intracellular protein-crowding sensor. A Förster resonance energy transfer (FRET) pair, involving a cyan fluorescent protein (CFP) insensitive to protein concentration and a glycine-inserted YFP, works as a genetically encoded probe to evaluate intracellular crowding. By measuring the fluorescence of the present FRET probe, we were able to detect dynamic changes in protein crowding in living cells.

Green fluorescent protein, which was the first fluorescent protein isolated from Pacific jellyfish (*Aequorea victoria*), is a popular and essential tool in biology because it allows simple and easy labelling^1^. Other colour variants have been constructed by direct mutagenesis and/or isolation from different species^2^,^3^, and probes to observe various intracellular physicochemical properties have been developed in conjunction with Förster resonance energy transfer (FRET) technology^4^,^5^,^6^. In general, the probes based on fluorescent proteins share a common problem for application as biosensors: fluorescent proteins possess an intrinsic sensitivity to solution conditions such as pH, chloride ion concentration, and temperature^7^,^8^,^9^,^10^. Looking at this problem from a different perspective, the fluorescent protein can be applied as a genetically encoded physicochemical sensor^7^,^10^,^11^,^12^. In this study, we tried to use another intrinsic sensitivity of fluorescent proteins in order to evaluate crowded conditions in living cells.

The crowded condition inside a cell is now an indispensable concept in cell biology. Goodsell’s “crowded cell” describes a cell that is more densely populated with proteins than with water molecules^13^. For example, the protein concentration inside an *Escherichia coli* cell was previously estimated to be approximately 350 mg/mL^14^,^15^, which is thought to cause micelle formation and aggregation of proteins^16^, and affects intracellular osmotic pressure^17^,^18^. The effects of such crowded conditions have been investigated in several molecular phenomena *in vitro*, including protein folding, enzymatic activity, and phosphorylation^19^,^20^. More recently, in-cell NMR spectroscopy studies have shown that protein folding in cells is significantly more stable than under *in vitro* conditions, suggesting that molecular crowding plays a major role in the stability of protein structure^21^,^22^,^23^. The molecular crowding effect is, at times, responsible for human amyloid disease such as Parkinson’s disease by accelerating the fibrillisation of α-synuclein, which is one of the components of Alzheimer’s disease amyloid^24^, and for the aggregation of fetal hemoglobin in sickle cell disease^25^.

The degree of molecular crowding directly relates to viscosity, which can be estimated from the diffusion coefficient determined using a simple probe such as a nanoparticle, organic dye, or fluorescent protein. Crowded conditions in cells, therefore, are currently evaluated by measuring the diffusion of those probes^26^,^27^,^28^,^29^. Because the crowding agents filling the cell are mainly proteins and nucleic acids, which are larger than small molecules such as polyethylene glycol and sucrose usually used in *in vitro* experiments as crowding agents^19^, protein diffusion *in vivo* is more complex than that *in vitro* for cases when the crowding agents form intracellular structures such as the cytoskeleton or nucleosomes. For example, protein diffusion in the nucleus depends on nucleosome dynamics, which facilitates the access of nuclear proteins to chromatin^28^. Additional information is, therefore, needed to evaluate intracellular crowded condition.

The density of the crowding agent in cells, e.g., protein concentration, is a valuable factor for evaluating intracellular crowding if it can be measured separately from protein diffusion. To directly measure protein mass in cells, a digitally recorded interference microscopy with automatic phase-shifting (DRIMPS) system was previously developed^30^. DRIMPS enables the measurement of whole protein mass in a living cell over the optical pathway, on the basis of the differences in refraction index. Raman microscopy also helps to study intracellular protein localization/distribution in a thin optical section^31^. Although these two optical methods are powerful evaluation tools for intracellular crowding because of the applicability to observe living cells, they cannot provide information about intracellular viscosity. To better estimate intracellular crowding using both protein density and viscosity, we developed a new fluorescent protein sensor based on FRET technology that enables simultaneous measurement of protein-based molecular crowding, called “protein crowding” in this paper, and measurement of protein diffusion by using fluorescence microscopy. The first part of this study shows the effect of protein crowding on various fluorescent proteins. We then describe the design, construction, and proof of concept of the genetically encoded probe for evaluating intracellular crowded conditions. In the last part, we show simultaneous measurement of diffusion coefficient and protein crowding by using this probe in living cells.

## Results

### Effect of protein concentration on fluorescent proteins

**Figure 1:**
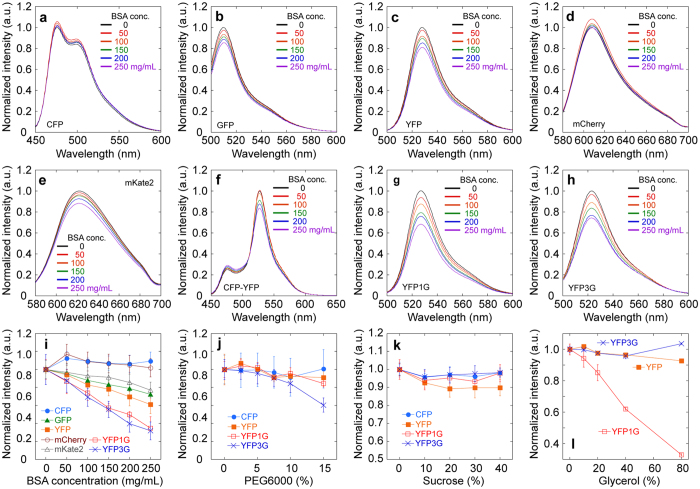
Effect of crowded conditions on fluorescent proteins. (**a–h**) Fluorescence of CFP (**a**), GFP (**b**), YFP (**c**), mCherry (**d**), mKate2 (**e**), CFP-YFP (**f**), YFP1G (**g**), and YFP3G (**h**) in the presence of 0–250 mg/mL BSA (*black*, 0 mg/mL; *red*, 50 mg/mL; *orange*, 100 mg/mL; *green*, 150 mg/mL; *blue*, 200 mg/mL; *purple*, 250 mg/mL). This fluorescence was recorded at the indicated wavelengths: CFP (**a**) at 450–600 nm; GFP (**b**), YFP (**c**), YFP1G (**g**), and YFP3G (**h**) at 500–600 nm; mCherry (**d**) and mKate2 (**e**) at 580–700 nm; and CFP-YFP (**f**) at 450–650 nm. The concentration of each fluorescent element was 0.01 mg/mL. (**i**) Effect of BSA concentration on CFP (c*yan filled circles*), GFP (*green filled triangles*), YFP (*orange filled squares*), mCherry (*brown open circles*), mKate2 (*grey open triangles*), YFP1G (*red open squares*), and YFP3G (*blue crosses*). (**j,k**) Effect of PEG6000 (**j**) and sucrose (**k**) on CFP (*cyan filled circles*), YFP (*orange filled squares*), YFP1G (*red open squares*), and YFP3G (*blue crosses*). (**l**) Effect of glycerol on YFP (*orange filled squares*), YFP1G (*red open squares*), and YFP3G (*blue crosses*).

First, we investigated the relationship between the concentration of proteins in solution and the fluorescence of various fluorescent proteins, including GFP, its cyan and yellow variants (CFP and YFP)^3^, the red fluorescent protein isolated from *Discosoma* species (mCherry)^32^, and the far-red fluorescent protein from *Entacmaea quadricolor* (mKate2)^33^. Bovine serum albumin (BSA) was selected for this assay because it is widely used as a standard protein for preparing calibration curves to measure protein concentration, such as in the Bradford method, and is a commonly used crowding agent that mimics the high protein concentration of living cells^19^,^20^. The fluorescence intensity of GFP (Fig. 1b), YFP (Fig. 1c), and mKate2 (Fig. 1e) decreased to ~80% relative to 0 mg/mL BSA in 250 mg/mL BSA solution, while that of CFP (Fig. 1a) and mCherry (Fig. 1d) slightly increased (Fig. 1i). The fluorescence intensities of CFP and YFP in the CFP-YFP pair conjugated with a flexible linker (GGSGGT), which is a widely used FRET pair^5^,^6^ showed an antiparallel response to BSA concentration (Fig. 1f). Thus, the intrinsic sensitivity of fluorescent proteins to protein crowding is a considerable problem similar to pH sensitivity.

We took advantage of this drawback of the intrinsic sensitivity of fluorescent proteins to protein crowding. We expected that the insertion of glycine residues before Tyr145, which altered the interaction between water molecules and the chromophore^34^, could enhance the sensitivity of YFP fluorescence to protein crowding. As expected, these mutant YFPs, named YFP1G (one glycine insertion) and YFP3G (three glycine insertion), showed higher dependence on BSA concentration than the original YFP (Fig. 1g–i). We next investigated the effect of two widely used distinct non-protein crowding agents on CFP, YFP, YFP1G, and YFP3G: polyethylene glycol (PEG6000) and sucrose. PEG6000 did not affect the fluorescence intensity of CFP, YFP, or YFP1G, whereas YFP3G showed enhanced dependence on PEG6000 (Fig. 1j). The fluorescence intensity of YFP decreased to 90% at >20% sucrose, which had no effect on CFP; interestingly, both glycine insertions did not enhance the sucrose dependence of YFP (Fig. 1k). On the other hand, glycerol, which is one of small polyols used as a viscosity agent, dramatically decreased YFP1G fluorescence, while YFP and YFP3G were insensitive at the concentrations tested (Fig. 1l). Although all three agents used here contribute toward increasing the viscosity of the solution, only glycerol affected YFP1G fluorescence. Considering the differences in the features of the three agents, hydrophobicity, rather than viscosity, is the reason for the dependence of YFP1G on BSA concentration.

To ascertain the cause of this BSA concentration dependence, we investigated the dependence of YFP1G on various other organic solvents. Most organic solvents we tested such as ethanol and methanol did not increase solution viscosity. The fluorescence intensity of YFP changed slightly, that of YFP1G simply decreased with increasing concentration, and the changes in YFP3G were more complex by the addition of organic solvents (Fig. S1). These results indicate that the origins of the protein-crowding sensitivity of YFP, YFP1G, and YFP3G differ, and that of YFP1G is the simplest and most likely, sensing the hydrophobicity of solution. Thus, the insertion of a glycine residue made YFP sensitive to solution hydrophobicity and enhanced crowding sensitivity. Additionally, the sensitivities of YFP1G to pH, chloride concentration, and other conditions, except the organic solution, were similar to those of wild-type YFP (Fig. S2). On the basis of its hydrophobic sensitivity, we chose YFP1G for detecting protein crowding.

### Construction of glycine-inserted mutant FRET (GimRET) probe as a protein-crowding sensor

**Figure 2:**
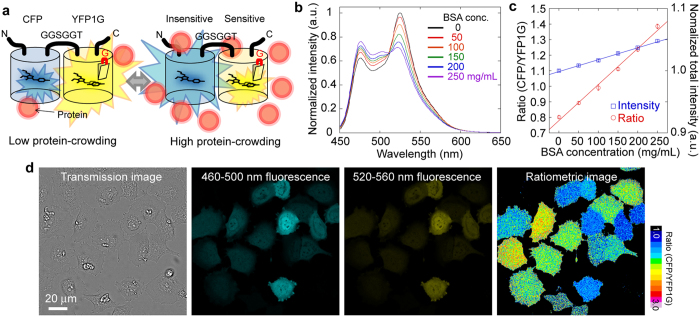
*In vitro* and intracellular observation of GimRET. (**a**) Schematic drawing of GimRET, which comprises the FRET pair CFP and YFP1G. (**b**) Fluorescence spectra of GimRET in the presence of 0–250 mg/mL BSA (*black*, 0 mg/mL; *red*, 50 mg/mL; *orange*, 100 mg/mL; *green*, 150 mg/mL; *blue*, 200 mg/mL; *purple*, 250 mg/mL). Traces represent the average of four trials. (**c**) Relationship between BSA concentration and the ratio of the total intensity observed at 460–500 nm and 520–560 nm (*red*) and the total intensity (*blue*). Plots represent the average of four trials. (**d**) Transmission image (*left*), fluorescence image at 460–500 nm (*left middle*), fluorescence image at 520–560 nm (*right middle*), and ratiometric image (*right*) of GimRET-expressing HeLa cells. Coloured bar indicates the ratio from 1.0 (*black*) to 3.0 (*white*).

A FRET pair of CFP and YFP1G that are insensitive and sensitive, respectively, to BSA concentration would form a genetically encoded probe to monitor intracellular protein crowding (Fig. 2a). The sensitivity of CFP-YFP1G fused with the GGSGGT linker to BSA concentration was enhanced (Fig. 2b,c) compared to that of CFP-YFP (Fig. 1f). To confirm the advantage of using the FRET phenomenon, we replaced the short linker to the longer one (GGSGGT × 6), to diminish FRET (Fig. S3), measured the fluorescence intensity of CFP at the excitation wavelength of 440 nm (Fig. S3a), and that of YFP1G at 488 nm (Fig. S3b), respectively, and plotted the ratio of them against the BSA concentration (Fig. S3c). Although the intensity ratio for CFP and YFP1G also correlated to BSA concentration without FRET, the percentage change in the ratio was ~3.0-folds less than that with the FRET, indicating the effectiveness of FRET in the ratiometry of CFP-YFP1G (Fig. S3d). We named the CFP-YFP1G GimRET (glycine-inserted mutant FRET probe). GimRET sensed both BSA concentration and that of other proteins including lysozyme, *E. coli* lysate, tubulin, actin, and nucleic acids (Fig. S4a–e). The dependence was unaffected by tubulin or actin polymerization (Fig. S4c,d). Non-specific binding of GimRET to the surrounding proteins, which affects diffusion^23^,^35^, could be neglected because GimRET fluorescence did not change above an ionic strength of 300 mM, which decreases non-specific protein binding (Fig. S4f). When investigating the correlation between the intensity ratio of the GimRET and the diffusion coefficient of the GimRET, by multiphoton fluorescence recovery after photobleaching (MP-FRAP)^36^, the intensity ratio responded only to BSA, but slightly to PEG6000 and sucrose, although all crowding agents decreased the diffusion mobility of GimRET, indicating the increase in solution viscosity (Fig. S5). This is an additional proof that the protein-crowding sensitivity of GimRET was derived not from viscosity, and in other words, GimRET senses selectively protein and/or nucleic acids crowding but not all of molecular crowding.

To confirm the applicability of GimRET to intracellular observations, we transfected the GimRET gene into mammalian cells (HeLa or Cos7 cells) and observed them under a multiphoton microscope. The cells showed heterogeneous expression of GimRET, and ratiometric images were obtained using the ratio of the intensity of CFP channel (460–500 nm) and YFP1G channel (520–560 nm) for each pixel (Fig. 2d and Fig. S6). We estimated the concentration of expressed GimRET to be 11.4 ± 8.9 μg/mL (Fig. S6c), from the correlation between the total intensity and the intensity ratio of CFP and YFP1G of GimRET (Fig. 2c, *blue*) and the calibration curve between the total intensity and the GimRET concentration *in vitro* (Fig. S7a). The intracellular concentration of GimRET in this range (1–100 μg/mL) did not affect the intensity ratio of CFP and YFP1G both microscopically and spectrophotometrically (Fig. S7b,c). The ratios were widely distributed (Fig. 2d, *right*, and Fig. S6d), indicating heterogeneous protein crowding because there was no correlation between CFP and YFP1G fluorescence intensity and their ratio (Fig. S6e).

**Figure 3:**
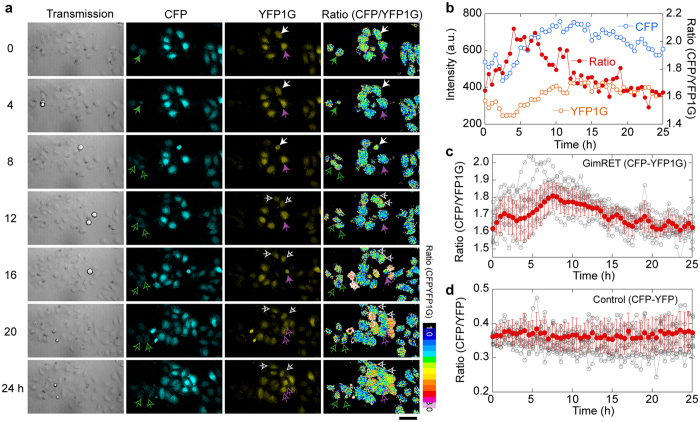
Live cell imaging of GimRET during cell division, by using a low-magnification objective lens. (**a**) Time-lapse transmission (*left*), fluorescence images at 460–500 nm (CFP, *left middle*), fluorescence images at 520–560 nm (YFP1G, *right middle*), and ratiometric images (*right*) of GimRET-expressing HeLa cells. Filled arrows, mother cells. Open arrows, daughter cells. Different cells are indicated with different colour arrows. Scale bar indicates 50 μm. Coloured bar indicates the ratio from 1.0 (*black*) to 3.0 (*white*). (**b**) Typical traces of the fluorescence intensity of CFP (*cyan*) and YFP1G (*orange*) and the intensity ratio (red) of a single cell just after cell division. (**c,d**) Ten typical traces (*grey*) and the average trace (*red*) of the intensity ratio of GimRET (**c**) and CFP-YFP (**d**) during the cell cycle. Error bars indicate standard deviation.

To confirm that GimRET can detect dynamic changes in intracellular protein crowding, we tracked the intensity ratio during cell division in GimRET-transfected HeLa cells (Fig. 3a, Fig. S8, and Movie S1). Because the intracellular protein concentration synchronizes with protein synthesis during the cell cycle due to an increased protein concentration at interphase, the intensity ratio of GimRET was expected to be synchronized with the cell cycle, increasing immediately after division and returning to normal levels thereafter. As expected, the intensity ratio of GimRET started to increase just after cell division, continued to increase for 5 h, and decreased to its initial value for 10 h (Fig. 3b and S8b). The cells that exhibited this increase-decrease intensity ratio behaviour were simultaneously observed in a field of view (Fig. 3c), unlike those expressing CFP-YFP as a control instead of GimRET (Fig. 3d). This dynamic change in protein concentration was similar to the findings of a previous study that measured the change in protein concentration in dividing yeast cells^37^. For further verification, we investigated the effects of the translation inhibitor cycloheximide^38^ and the proteasome inhibitor MG132^39^. Cycloheximide inhibited the increase of the intensity ratio, while MG132 promoted it (Fig. S9a–d). Protein concentration is thought to exponentially decrease from the first-order protease reaction after cycloheximide addition, but to linearly increase from protein synthesis after MG132 addition, and then gradually saturate because of lack of material. The behaviour of GimRET likely reflected these conditions (Fig. S9e). CFP-YFP also exhibited a changed ratio, but the change was small relative to that of GimRET (Fig. S9f). These results support the view that GimRET is capable of measuring temporal changes in protein crowding in living cells.

### GimRET and pH during cell volume changes

**Figure 4:**
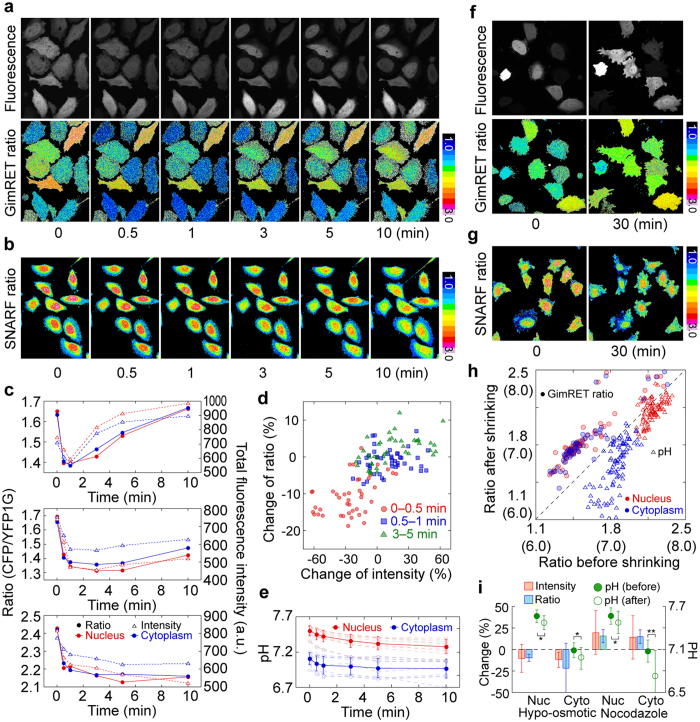
Comparison of the GimRET ratio and intracellular pH obtained by SNARF-1 in swelling and shrinking cells. (**a,b**) Time-lapse fluorescence images of the total fluorescence intensity (**a**, *upper*), the ratio of 460–500 nm and 520–560 nm (a, *lower*) in GimRET-expressing HeLa cells, and the ratio of 640–700 nm and 500–560 nm in the HeLa cells labelled with SNARF-1 (**b**) on addition of a hypo-osmotic medium. (**c**) Three typical traces of the intensity ratio (*filled circles*) and total intensity (*open triangles*) in the nucleus (*red*) and cytoplasm (*blue*) after hypo-osmotic treatment. (**d**) Correlation between the percentage changes in the total intensity and intensity ratio at 0–0.5 min (*red*), 0.5–1.0 min (blue), and 3–5 min (*green*). Each single dot indicates a single cell. (**e**) Ten typical traces of the intracellular pH estimated from the SNARF-1ratio (*broken lines*) and average traces (*solid lines*) in the nucleus (*red*) and cytoplasm (*blue*) after adding the hypo-osmotic medium. Error bars indicate standard deviation. (**f,g**) Fluorescence images of the total fluorescence intensity (**f**, *upper*), the ratio of 460–500 nm and 520–560 nm (**f**, *lower*) in HeLa cells expressing GimRET, and the ratio of 640–700 nm and 500–560 nm in HeLa cells labelled with SNARF-1 (**g**) before (*left*) and 30 min after (*right*) adding 10 μM nocodazole. (**h**) Correlation between the GimRET ratio (*filled circles*) and intracellular pH (*open triangles*) in the nucleus (*red*) and cytoplasm (*blue*) before and 30 min after adding nocodazole. Each single dot indicates a single cell. (**i**) Graph summarizing the changes before and after the hypo-osmotic (1 min) and nocodazole treatments of the total intensity (*red bars*), the GimRET ratio (*blue bars*), and the pH (*green circles* (*filled*, before; *open*, after)). Single and double asterisks correspond to P value is respectively < 0.05, and <0.01 in two sample t-test.

pH sensitivity is a common problem in the development of functional FRET probes based on fluorescent proteins^5^,^6^. Because GimRET is also affected by pH (Fig. S10), we considered whether intracellular pH or protein crowding predominantly influenced changes in its fluorescence. We investigated the relationship between GimRET fluorescence and intracellular pH over changes in the cell volume on addition of a hypo-osmotic medium^40^. After the cells were swollen by adding a hypo-osmotic medium, the total fluorescence intensity of CFP and YFP1G transiently decreased by 10–20% within 1 min, and then gradually returned to its initial values in the following 9 min (Fig. 4a, *upper*, and Fig. 4c, *upper and middle, open triangles*). Some cells exhibited a unidirectional decline in fluorescence intensity, indicating slow swelling (Fig. 4c, *lower, open triangles*). Although the change in ionic strength might be induced by the addition of the hypo-osmotic medium, it is highly unlikely that the change in ionic strength impacted the fluorescence intensity of GimRET, because the fluorescence intensity of GimRET is quite stable under an ionic strength of 300 mM (Fig. S4f) and because glycine insertion did not affect the sensitivity of YFP to various cations (Fig. S2). Additionally, new fluorescent protein cannot be synthesized nor degraded on this time scale in HeLa cells (synthesis rate = 0.032 h^−1^ and degradation rate = 0.032 h^−1^, Fig. S11). The rapid decrease and slow recovery of fluorescence intensity observed here was consistent with the changes in cell volume induced by hypo-osmotic pressure, as previously reported^40^. Therefore, the change in total fluorescence intensity is due to the changes in cell volume. The change in intensity ratio positively correlated with that in total intensity (Fig. 4a, *lower*, and Fig. 4c, *filled circles*), and the percentage change of the ratio correlated to that of intensity (Fig. 4d), indicating that GimRET could monitor intracellular protein crowding along with the volume change of cells. On the other hand, the intracellular pH estimated with an organic pH indicator, namely, SNARF-1^41^, unidirectionally decreased in all cells (Fig. 4b,e). The change in the intensity ratio of GimRET was opposite to its pH sensitivity: the intensity ratio would increase if GimRET sensed pH changes. These data indicates that GimRET can detect changes in protein crowding, rather than changes in the pH value.

The opposite reaction in the swelling assay was a further indication that GimRET detects changes in intracellular protein crowding. We observed changes in GimRET intensity when the cell volume was reduced by disrupting the cytoskeleton with nocodazole, an inhibitor of microtubule polymerization. Thirty minutes after adding 10 μM nocodazole, the total fluorescence intensity of CFP and YFP1G increased, indicating that the cell had shrunk, and the intensity ratio increased, indicating greater protein crowding (Fig. 4f,h, *filled circles*, and Fig. 4i, *red and blue*). The pH of both nucleus and cytoplasm decreased (7.6 to 7.5 in nucleus; 7.1 to 6.7 in cytosol), similar to the changes observed in the swelling assay (Fig. 4g,h, *open triangles*, and Fig. 4i, *green*). The response of GimRET to varying intracellular pH (Fig. S12) predicted the intensity ratio to decrease by 6% in the nucleus and 4% in the cytoplasm after nocodazole treatment. Since the change on adding nocodazole was ~23%, at least 17% of the ratio change was likely to be derived from the changes in intracellular protein crowding. Changes in the intensity ratio of CFP-YFP were different from those observed for GimRET: the intensity ratio simply decreased in swelling cells, but it did not change in shrinking cells (Fig. S13). Furthermore, we confirmed the correlation between the decrease in cell volume and the increase of GimRET ratio by using an osmotic shock assay using *E. coli* cells (Fig. S14).

**Figure 5:**
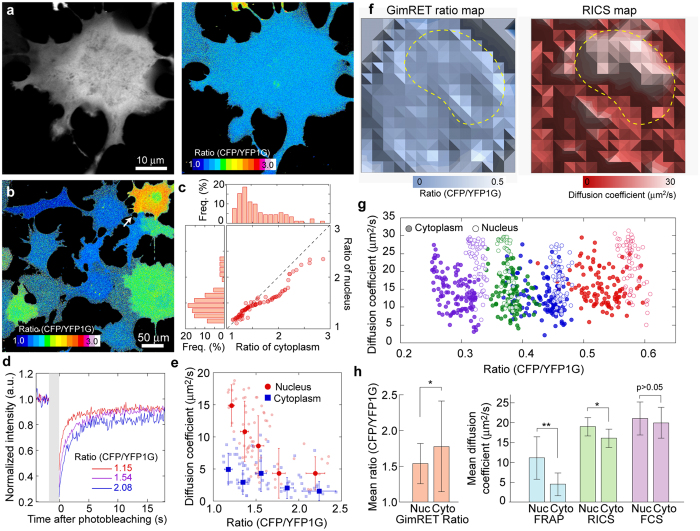
Relationship between the GimRET ratio and protein diffusion. (**a**) High-magnification fluorescence (*left*) and ratiometric (*right*) images of Cos7 cells transfected with GimRET. (**b**) Low-magnification ratiometric image of Cos7 cells transfected with GimRET. Colour bars indicate the ratio from 1.0 (*black*) to 3.0 (*white*) in (a) and (b). (**c**) Relationship of the ratios from the cytoplasm and nucleus (*right lower*) and histograms of the ratios from the cytoplasm (*upper*) and nucleus (*left*). The broken line indicates a 1:1 correlation. N = 91 cells. (**d**) Photorecovery curves of FRAP measurement inside the nuclei with ratios of 2.08 (*blue*), 1.54 (*purple*), and 1.15 (*red*). (**e**) Relationship between the ratio and diffusion coefficient from FRAP measurement in the cytoplasm (*blue*) and nucleus (*red*) N = 69 cells. Error bars indicate standard deviation. Light-coloured symbols indicate individual cells. (**f**) GimRET ratio map (*left*) and 15 × 15 RICS diffusion map (*right*). Coloured bars indicate the ratio from 0 (*blue*) to 0.5 (*white*) and 0 (*red*) to 30 (*white*), respectively. Broken lines indicate the boundary between the nucleus and cytoplasm. The image width/height is 22.5 μm (one pixel = 1.5 μm). (**g**) Relationship between the ratio and RICS diffusion coefficient in the nucleus (*open*) and cytoplasm (*filled*) of four cells. Each colour indicates a single cell. (**h**) Mean values of the intensity ratio (*red*) and diffusion coefficients obtained by FRAP (*blue*), RICS (*green*), and FCS (*purple*) in the nucleus (*Nuc*) and cytoplasm (*Cyto*). N = 71, 71, 71, 71, 11, 11, 18, and 14 cells, respectively. Error bars indicate standard deviation.

Comparison of the ratiometric images of GimRET and SNARF-1 also suggested that GimRET responds to protein crowding. The intensity ratio of GimRET was almost the same for both nucleus and cytoplasm, which underwent obvious different pH changes (Fig. 4a,b,i). Furthermore, Cos7 endosomes showed low pH (Fig. S15) and Neuro 2A mitochondria showed high pH (Fig. S16a), but GimRET fluorescence in these cells as well as in HeLa cells did not depend on the subcellular location (Fig. 5a and Fig. S16b). Therefore, GimRET detected changes in protein crowding rather than in intracellular pH, despite its pH sensitivity.

### Relationship between GimRET intensity ratio and protein diffusion

Crowded conditions in cells are usually evaluated by measuring the diffusion of probes such as a nanoparticle, organic dye, or fluorescent protein^26^,^27^,^28^,^29^. When observing GimRET-expressing Cos7 cells, we found that the distribution of GimRET intensity ratio in both cytoplasm and nucleus was almost uniform, predicting slight dependence of the diffusion coefficient of a probe on a local site in the cytoplasm or the nucleus (Fig. 5a), as previously demonstrated^29^,^42^,^43^. The cells showed different intensity ratio of GimRET, and the intensity ratio of the cytoplasm was nearly equivalent to that of the nucleus in many cells (Fig. 5bc), but not in all cells (Fig. 5b, *white arrow*).

We compared the intensity ratio and diffusion coefficient measured by MP-FRAP (Fig. S17). The intensity ratio of GimRET correlated well with the diffusion coefficient of the cells (Fig. 5d,e). Interestingly, the correlation between the intensity ratio and diffusion coefficient in each cellular region was different (Fig. 5e). The effect of nuclear transport was negligible, because the nuclear import/export rates of GimRET were much slower than the free diffusion of GimRET measured with the same method (Fig. S18; import rate, 164 s; export rate, 197 s). For CFP-YFP, different ratios in the nucleus and cytoplasm and the correlation between the intensity ratio and diffusion coefficient were not observed (Fig. S19). Furthermore, the intensity ratio of the cells did not vary so much: the variation coefficients of CFP-YFP were 0.07 for nucleus and 0.08 for cytoplasm (Fig. S19e), while those of GimRET were 0.16 and 0.22 (Fig. 5c), respectively. Therefore, GimRET and CFP-YFP exhibit different behaviours in living cells.

We also compared GimRET ratiometry and raster image correlation spectroscopy (RICS) (Fig. 5f, and S20), a method to obtain a diffusion map^44^,^45^, because diffusion coefficient estimation depends on the measurement method^46^,^47^,^48^. By binning the intensity ratios into a small space of 1.5 μm^2^, the small spatial dependence of GimRET ratio could be observed (Fig. 5f, *left* and Fig. S20, *middle*). The RICS provides the diffusion coefficient in the same square (Fig. 5f, *right* and Fig. S20, *right*). There was a rough, but certain, correlation between the diffusion coefficient and intensity ratio of GimRET in a single cell (Fig. 5g). The tendency that mean diffusion coefficient in the nucleus is larger than that in the cytoplasm was consistently observed in the methods tested by us, including fluorescence correlation spectroscopy (FCS)^42^,^43^,^49^, and GimRET exhibited greater protein crowding in the cytoplasm than in the nucleus in the mean (Fig. 5h). Thus, GimRET gives us information about protein crowding, in addition to viscosity.

## Discussion

In this study, we showed the sensitivity of fluorescent proteins to protein crowding and the unconventional approach of harnessing of this sensitivity to develop a genetically encoded protein-crowding sensor. The fluorescence of the fluorescent proteins tested by us, namely, YFP, GFP, and mKate2, was dependent on BSA concentration. This dependence has to be considered when using these fluorescent proteins as an intracellular probe.

We successfully enhanced the protein-crowding dependence of YFP by inserting a glycine residue into it. Based on our investigation of YFP1G dependence on various factors, GimRET sensitivity to protein crowding is thought to result from the hydration of proteins and nucleic acids. The correlation between the hydrophobicity of the solution and protein concentration was confirmed using a fluorescence solvatochromic dye, POLARIC (Fig. S21)^50^. GimRET responded to concentration, but not to the polymerization of tubulin and actin (Fig. S4c,d). The reactivity of GimRET to protein concentration depended on the protein species: the percentage changes per 1 mg/mL BSA and lysozyme, which are relatively soluble in water, are 0.17% and 0.38%, respectively, while those per 1 mg/mL actin and tubulin, which are less soluble in water, were larger, that is, 2.2% and 3.1%, respectively. Moreover, *in vitro* MP-FRAP clearly showed the independence of GimRET ratio to solution viscosity (Fig. S5). We, therefore, concluded that GimRET certainly senses crowded conditions in its surroundings, primarily derived from proteins and nucleic acids, via sensitivity to hydrophobicity.

The mechanism of YFP1G protein crowding sensing is highly complex and could not be conclusively determined in this study; however, the absorbance spectra provided a clue regarding the sensing mechanism. The absorbance of YFP did not decrease with the addition of BSA, while that of YFP1G did (Fig. S22). The surrounding protein thus decreased the quantum yield of YFP fluorescence, and the molar absorption coefficient of YFP1G decreased because of the static interactions between the chromophore and water molecules^51^. The static water interaction was also previously observed through the analysis of crystal structures^34^. A comparison of the crystal structure of the periphery of YFP1G chromophore and the original YFP showed that glycine insertion before Tyr145 flipped a part of the β-sheet composed of Tyr145 to His149 toward the outer side of the β-can structure (Fig. S23), producing the access pathway for water molecules between the chromophore and external environment. Excess solution might affect the accessibility of water molecules to the inner side of the β-can structure via the generated pathway. Water molecules in the solution tend to be trapped in the hydrophilic regions. When the water molecules are absorbed by protein hydration at high protein concentrations, the inner environment of YFP1G may become more hydrophilic than the surface, resulting in static water molecule localization near the chromophore.

The major mechanism of the effect of molecular crowding can be described in terms of excluded volume effect^20^. The excluded volume effect exerted by molecular crowding might alter the structure and/or folding of fluorescent proteins, causing changes in their fluorescence. Because the spectral peak of YFP is red-shifted than that of GFP own to the contribution of the π-π stacking present between the GFP chromophore and the phenol ring of the Thr203^52^, an extra perturbation to the structure of YFP induces peak shift in the fluorescence spectrum^53^. The spectral peak of YFP1G, therefore, would shift if the YFP structure was altered by the excluded volume effect. By increasing the percentage of glycerol in the solution, we found that the spectral peak of YFP1G greatly shifted toward blue at higher concentrations (>40%), but slightly at lower concentrations (Fig. S24b,d). YFP3G showed a gradual blue-shift, whereas YFP showed no spectral shift (Fig. S24a,c,d). The high BSA concentration also caused the blue-shift of YFP3G, but not of YFP or YFP1G (Fig. S24e). Protein crowding primarily induced changes in the fluorescence intensity of YFP1G (Fig. 1l). Therefore, the excluded volume effect exerted on YFP1G is thought to have caused slight changes in the chromophore.

We observed differences in the intensity ratio of GimRET and the intracellular pH estimated by SNARF-1 (Fig. 4) and confirmed that photobleaching, which is another consideration when using FRET probes, was negligible in our experimental setup (Fig. S25). Therefore, the ratiometric changes of GimRET presented here primarily originate from the sensitivity of YFP1G to protein crowding and not from the intracellular pH or photobleaching. However, the users of GimRET should be aware of its pH dependence because the intensity ratio changes by 10–15% when the intracellular pH is decreased from 7.5 to 6.7 or increased to 7.7 in the nucleus (Fig. S12). The applicability of GimRET is limited to conditions with stable intracellular pH, similar to other fluorescent protein probes^5^,^6^. We recommend the users of the fluorescent protein probes, including GimRET, to ensure that the intracellular pH is maintained stable during the observation period, by using a pH indicator such as SNARF-1. Another important factor is Cl^−^ dependence. Although the spectrum is affected by the presence of Cl^−^, the intensity ratio is relatively stable because YFP1G and CFP have similar Cl^−^ dependences at high protein concentrations (Fig. S26).

On transfecting GimRET into mammalian cells, the cells showed heterogeneous expression of GimRET (Fig. 2d and S6). The distribution of the expression was expected to be much narrower if the heterogeneity was induced by intracellular pH: the intracellular pH of Cos7 cells was estimated to be 7.6 ± 0.1 in the nucleus and 7.5 ± 0.1 in the cytoplasm by SNARF-1 (Fig. S12d), and the predicted GimRET ratios were 1.8 ± 0.1 and 1.7 ± 0.1, respectively (Fig. S12e). We should be concerned about the possibility that the heterogeneous GimRET ratio among cells may be derived from the differences in the folding/maturation rate between the donor and accepter probes in the fluorescent protein-based FRET, which yields erroneous intensity ratios. The maturation rate of YFP is slower than that of CFP, and glycine insertion did not affect the maturation rate in *E. coli* (Fig. S27a). This difference affects the ratio in CFP-YFP (Fig. S27b). Interestingly, YFP1G in GimRET matured faster than YFP1G alone, and this reduced the error in intensity ratio (Fig. S27c). The saturation rate of the intensity ratio was estimated to be ~0.7 h^−1^, which was 20-fold faster than the rates of synthesis and degradation (~0.03 h^−1^, Fig. S11). Therefore, it is unlikely that the folding/maturation rate affected the calculation of the intensity ratio of GimRET. In addition, the diffusion coefficient obtained by FRAP correlated to the GimRET ratio (Fig. 5e). It can be thus concluded that the variability of GimRET ratio among cells was due to the heterogeneity of intracellular protein crowding.

To confirm that GimRET holds credit in crowded cellular conditions as a protein-crowding sensor, we observed the protein distribution in cells by using Raman microscopy that allows us to extract the spectral peaks indicating proteins^31^,^54^. The images reconstructed based on the peak intensity indicating the cellular proteins (1686 cm^−1^) showed no organelle-specific localization, similar to the ratiometric image offered by GimRET (Fig. S28). This distribution was also observed in a previously developed method that provides a viscosity map for cells^29^. On the other hand, differences among the methods of measuring diffusion coefficients have been debated^46^,^47^,^48^, and the present study also shows these differences (Fig. 5). The observation that mean diffusion in the nucleus is faster than that in the cytoplasm was consistent in all methods tested by us (MP-FRAP, RICS, and FCS), and GimRET exhibited greater protein crowding in the cytoplasm than in the nucleus in the mean. Because the intensity ratio of GimRET correlated to the RICS diffusion coefficient in single cells, the GimRET ratio can be considered to reflect rapid diffusion, and several factors such as the cytoskeletal scaffold and small lipids can cause slower protein diffusion in the cytoplasm than in the nucleus. The inability of GimRET to experimentally detect changes in the protein state/structure contributes to the mismatch between the GimRET ratio and diffusion coefficient because protein diffusion is affected by the state/structure of surrounding proteins. The advantage of using GimRET is to simultaneously provide two kinds of information responsible for the crowded condition: protein density by ratiometry and surrounding viscosity by diffusion measurement. We propose that measuring the fluorescence intensity ratio of GimRET in combination with estimating its diffusion coefficient by several methods is necessary to evaluate intracellular crowded conditions.

Another type of FRET sensor for macromolecular crowding was most recently developed based on structural changes in an artificially designed hinge structure peptide composed of two α-helices and one flexible linker which change conformation depending on the crowded condition^55^. GimRET does not use this type of functional peptides; however, the acceptor changes its fluorescence intensity with protein crowding, while the donor remains inert. Interestingly, that sensor responded to PEG concentration, while GimRET did not. It is most likely that these two probes predominantly sense the distinct components constituting the crowded condition. Because there are many components of intracellular crowding, the parallel use of two probes might enable a more precise evaluation of the intracellular crowded conditions.

## Conclusion

To summarize, we describe the intrinsic sensitivity of fluorescent proteins to protein crowding, and the novel FRET probe, namely, GimRET, which can measure protein crowding inside living cells. Although further investigation of the physical mechanism underlying the effects of glycine insertion and further improvement of GimRET is necessary, GimRET has the potential to function as an environmental indicator.

## Materials and Methods

### Gene construction and purification of fluorescent proteins

The expression vectors pECFP-C1, pEGFP-C1, and pmCherry-C1 were purchased from Takara-Clontech (TAKARA, JP), and pmKate2-C was purchased from evrogen (evrogen, RU). EYFP was constructed by substituting four EGFP residues: Ser-65 to Gly, Val-68 to Leu, Ser-72 to Ala, and Thr-203 to Tyr, as previously reported^52^. The cDNAs of YFP-1G and YFP-3G were obtained as PCR products by using primers including G and GGG, respectively^34^. The cDNAs of these fluorescent proteins were amplified by PCR using sense primers containing a NdeI site and reverse primers containing a HindIII site; the PCR products were ligated into the *E. coli* expression vector pAL7 (BIO-RAD, USA) between the NdeI and HindIII sites for plasmid construction and transformed into the *E. coli* variants DH5α or Rosseta2 (DE3) (Merck Millipore, DE). For FRET probe construction, the cDNA of YFP or YFP1G was amplified by PCR using sense primers containing the sequence encoding the flexible linker (GGSGGT) and C-terminal sequence of CFP containing the BspEI site and reverse primers containing the XhoI site. The PCR product was ligated into a pECFP-C1 vector between the BspEI and XhoI sites and then transformed into DH5α. The NheI/NcoI fragment, including YFP1G and CFP, was ligated into a pAL7 vector between the NheI and NcoI sites and transformed into Rosseta2.

Profinity eXact^TM^ Fusion-Tag system (BIO-RAD, USA) was used to purify tag-free proteins containing the native N-terminal amino acid sequence. About 4.0–5.0 mg of protein was obtained. After purifying the fluorescent proteins, we concentrated them to 10 mg/mL and changed the buffer to 1 mM HEPES (pH 8.0) by using Amicon Ultra Centrifugal Filters (Merck Millipore, DE).

### Measurement of fluorescence and absorbance spectra

We adjusted the pH after dissolving BSA in 100 mM HEPES buffer (pH 8.0, 7.4, and 7.0) or 100 mM MES buffer (pH 6.5 and 6.0). PEG6000 or sucrose was dissolved into 100 mM HEPES (pH 7.4). Glycerol, ethanol, or the other organic solvent was diluted using 100 mM HEPES (pH 7.4). Lysozyme chloride was dissolved into 100 mM HEPES (pH 7.4), and then the solution was dialyzed to remove chloride ions.

The fluorescent proteins were diluted to 0.01 mg/mL in each solution and then scanned for fluorescence (RF5300-PC fluorescence spectrophotometer; Hitachi, JP). The excitation wavelength was set to 440 nm for CFP, GimRET, CFP-YFP, and long-linker GimRET; 488 nm for GFP, YFP, and YFP variants; and 530 nm for mKate2 and mCherry. We scanned the following wavelength ranges for emission pattern: 450–600 nm for CFP; 500–650 nm for GFP, YFP, and YFP variants; 580–700 nm for mKate2 and mCherry; and 450–650 nm for GimRET, CFP-YFP, and long-linker GimRET. For measuring absorbance spectra, the solution was scanned for absorbance between 250 and 600 nm (UV-Vis Spectrophotometer UV-1650PC, Shimadzu, JP).

### Cell-line culture and transfection

HeLa cells (human epithelial carcinoma cell line), Cos7 cells (African green monkey kidney-derived cell line), and Neuro2A cells (mouse neural crest-derived cell line) were purchased from Riken Cell Bank (Tsukuba, JP), and checked for mycoplasma contamination using mycoplasma PCR detection kit (e-Myco™ plus; iNtRON, KR). Cells were cultured in Dulbecco’s modified Eagle’s medium (DMEM; Sigma-Aldrich, MO, USA) supplemented with 10% fetal calf serum (Sigma-Aldrich, USA) and 1% Penicillin-Streptomycin (Sigma-Aldrich, MO, USA), under 5% CO_2_ at 37 °C. Cells were trypsinized 24 h before transfection and replated onto collagen-I coated cover slips (IWAKI, JP). Transient transfections were performed with Fugene-HD (Promeda, USA), according to the manufacturer’s instructions. The medium was replaced with phenol red-free medium to wash out Fugene-HD agents, 24 h after transfection.

### Measuring GimRET expression in cells by using multiphoton fluorescence microscopy

Multiphoton fluorescence experiments, including imaging and MP-FRAP, were performed using an inverted multiphoton laser scanning microscope (FV1000-MPE; Olympus, JP) combined with an incubator for stable cell culture (CytoGROW^TM^ GLP; Sanyo, JP). We used a 25× objective lens (NA 1.05, water, XLPLN; Olympus, JP) for low-magnification observation and 60× objective lens (NA 1.49, oil, PLAPON; Olympus, JP) for high-magnification observation and MP-FRAP. Using the 25× objective lens, the observation area was 508.43 × 508.43 μm^2^ (1024 × 1024 pixels), and the pixel dwell time was 0.497 μs. Using the 60× objective lens, the observation area was 211.76 × 211.76 μm^2^ (1024 × 1024 pixels), and the pixel dwell time was 0.207 μs. The fluorescence images were averaged five times. The excitation wavelength was 880 nm, and the fluorescence of GimRET or CFP-YFP was separated into that of CFP and that of YFP1G or YFP by a dichroic mirror (505 nm) and filtered by corresponding band-pass filters (460–500 nm and 520–560 nm). Data analysis was performed using Image-J. The fluorescence intensity was measured after background subtraction. The intensity ratio was calculated by dividing the fluorescence intensity of CFP with that of YFP1G or YFP.

We measured MP-FRAP using a previously established procedure^36^. The experimental condition is described in the legend of Fig. S17. Because the diffusion coefficient depends on the estimation method^56^, we estimated them with the calibration curve of the diffusion coefficient *D* per the time constant *k*. We measured the *k* values of fluorescent latex beads (φ = 20 μm) in glycerol solution (20–80%), and obtained the correlation of the *k* and the theoretically calculated *D*^57^.

The image data was analysed using Image-J or a homemade software written in Visual Studio 2005 (Microsoft, WA).

### Raster image correlation spectroscopy (RICS)

Laser raster scanning images of GimRET-expressing Cos7 cells were acquired by LSM 510 META-ConfoCor3 (Carl Zeiss, DE), with a pixel dwell time of 51.2 μs, a frame size of 2048 × 2048 pixels, and a pixel size of 0.011 μm. The confocal pinhole diameter was adjusted to 70 μm. GimRET was excited at 405 nm. The emission signals were split by a dichroic mirror (NFT515, Zeiss, DE) and detected at 420–475 nm for CFP and 530–575 nm for YFP. The time series of five images were recorded. RICS analysis was performed using an internal software, written in MATLAB (MathWorks, USA). To filter out the immobile features of the images, the centre image of time series was subtracted with the average image of the entire image stack, pixel by pixel, and added to the average raw image intensity as a scalar. The spatial autocorrelation function *G* (*ξ*, *ψ*) was computed from the processed image^45^,^58^,^59^:


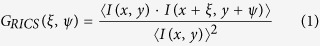


where *I*(*x*, *y*) is the intensity of the calculated image at the coordinates (*x*, *y*), *ξ* and *ψ* are the spatial increments in x and y, respectively. *G*_RICS_ (*ξ*, *ψ*) was fitted to the diffusion model:


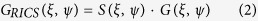



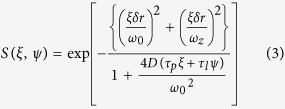



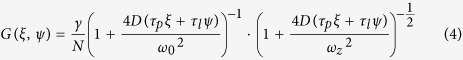


where *S* (*ξ*, *ψ*) and *G* (*ξ*, *ψ*) are the correlation functions due to laser beam scanning and diffusion, respectively. The pixel size δr, the pixel dwell time τ_*p*_ and the line repetition time τ_*l*_ were determined by the condition of the microscopy. ω_*0*_ and ω_*z*_ are the width and height, respectively, of the confocal volume (PSF), whose values were determined by FCS. γ is the shape factor, which was fixed at 1.0 in this study, as the value of the FCS model^60^. The average number of particles in the confocal volume (*N*) and diffusion coefficient (*D*) are derived from fitting analysis. The value of *G*_RICS_ (0, 0) was excluded from fitting because it contained large shot noise. In this study, the laser scanning speed in the y direction was 4096 (= 2 × *x* resolution) times slower than that in *x* direction. This is very slow, compared to the molecular diffusion of samples. Therefore, only *x* cross-sections of the autocorrelation functions (ACFs) *G*_RICS_ (*ξ*, 0) were analyzed. A 2D map of diffusion coefficient distribution (diffusion map) was generated using a sub-region of 256 × 256 pixels with 128 pixel shifts in the *x* and *y* directions from the detrended image.

### SNARF observation in living cells

Before observation, the cells were incubated in the medium containing 5 μM SNARF-AM acetate (Thermo Fisher Scientific, USA). The medium without phenol red was replaced 30 min after incubation to wash out SNARF-AM and then the cells were observed under a multiphoton laser scanning microscope at 37 °C. SNARF was illuminated by a green laser (473 nm) (FV10-LD473; Olympus, JP), and the fluorescence was separated by a dichroic mirror (560 nm) and detected using a spectrophotometer (FV10-SPD; Olympus, JP) between 500 and 560 nm and 640 and 700 nm. The intensity ratio was calculated by dividing the fluorescence intensity between 640 and 700 nm and 500 and 560 nm after background subtraction.

### Forced cell volume change

Cells expressing GimRET or CFP-YFP or stained with SNARF were incubated in 20 mM phosphate-buffered saline (PBS; pH 7.4) with 150 mM sucrose for 10 min before observation, and then observed using fluorescence microscopy. When expanding the cells, sucrose concentration in the medium was changed from 150 to 75 mM by adding a hypo-osmotic medium (20 mM PBS, pH 7.4), during microscope observation, to swell the cells. The time points of observation were as follows: before the addition of the hypo-osmotic medium, and then 0.5, 1, 3, 5, and 10 min after the addition of the medium. When shrinking cells, the cells were observed in DMEM, and incubated for 30 min on the microscope to shrink the cells by changing the medium to that containing 10 μM nocodazole (WAKO, JP) and, and then observed the same cells.

## Additional Information

**How to cite this article**: Morikawa, T. J. *et al.* Dependence of fluorescent protein brightness on protein concentration in solution and enhancement of it. *Sci. Rep.* **6**, 22342; doi: 10.1038/srep22342 (2016).

## Supplementary Material

Supplementary Movie S1

Supplementary Figures
