# Supplementary material for: Dependence of fluorescent protein brightness on protein concentration in solution and enhancement of it
Source: Sci Rep. 2016 Mar 9;6:22342. doi: 10.1038/srep22342 (PMC4783657; doi:10.1038/srep22342)
Supplement: Supplementary Figures [file srep22342-s2.pdf]

Dependence of fluorescent protein brightness on protein concentration in solution and enhancement of it.

Takamitsu J. Morikawa[1], Hideaki Fujita[2,4], Akira Kitamura[5], Takashi Horio[5], Jotaro Yamamoto[5], Masataka Kinjo[5], Akira Sasaki[6], Hiroaki Machiyama[2], Keiko Yoshizawa[4], Taro Ichimura[4], Katsumi Imada[7], Takeharu Nagai[3], *Tomonobu M. Watanabe[1,2,4,8]

[1]Graduate School of Frontier Bioscience, [2]WPI, Immunology Frontier Research Centre, and [3]Institute of Scientific and Industrial Research Centre, Osaka University, Suita, Osaka 565-0871, JAPAN; [4]RIKEN Quantitative Biology Centre (QBiC), Suita, Osaka 565-0874, JAPAN; [5]Laboratory of Molecular Cell Dynamics, Faculty of Advanced Life Science, Hokkaido University, Sapporo 001-0021, JAPAN; [6]Biomedical Research Institute, National Institute of Advanced Industrial Science and Technology (AIST), Tsukuba, Ibaraki 305-8566, JAPAN; [7]Department of Macromolecular Science, Graduate School of Science, Osaka University, Toyonaka, Osaka 565-0043, JAPAN; [8]PRESTO, Japan Science and Technology Agency, 4-1-8 Honcho Kawaguchi, Saitama 332-0012, JAPAN

Supplemental Figures

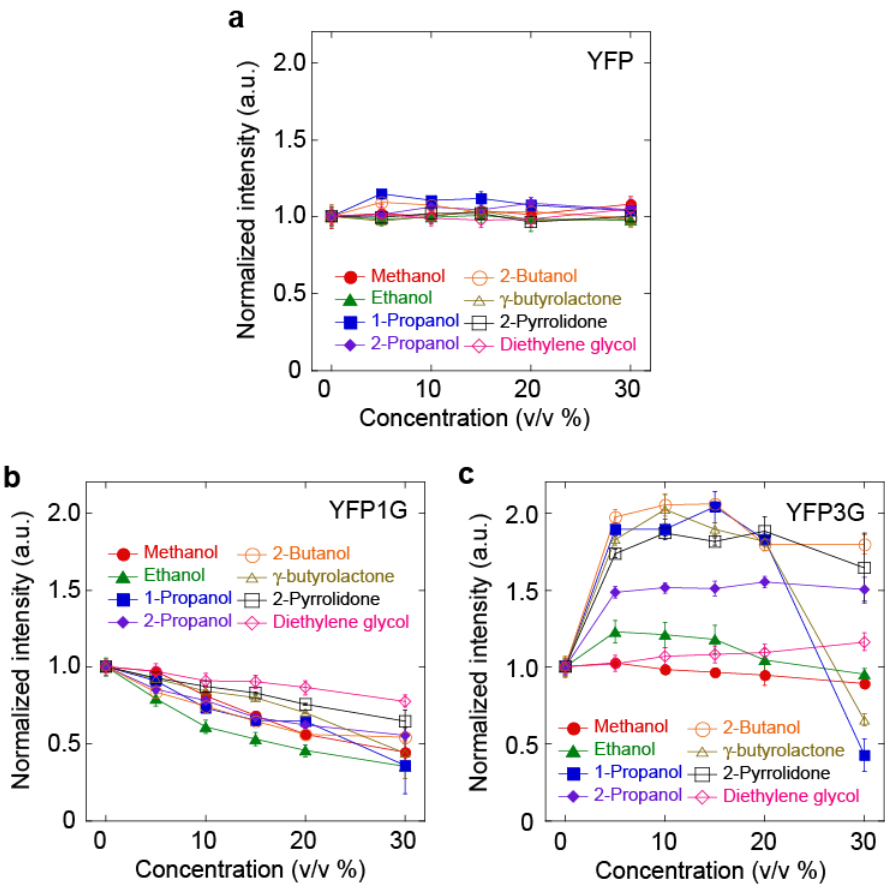

Figure S1. Dependences of YFP and glycine-inserted mutants on hydrophobicity.

Concentration dependences of YFP (**a**), YFP1G (**b**), and YFP3G (**c**) on methanol (*red filled circles*), ethanol (*green filled triangles*), 1-propanol (*blue filled squares*), 2-propanol (*purple filled rhombi*), 2-butanol (*orange open circles*), γ-butyrolactone (*ochre open triangles*), 2-pyrrolidone (*black open squares),* and diethylene glycol (*pink open rhombi*). All plots represent the average of four trials. Error bars, standard deviations.

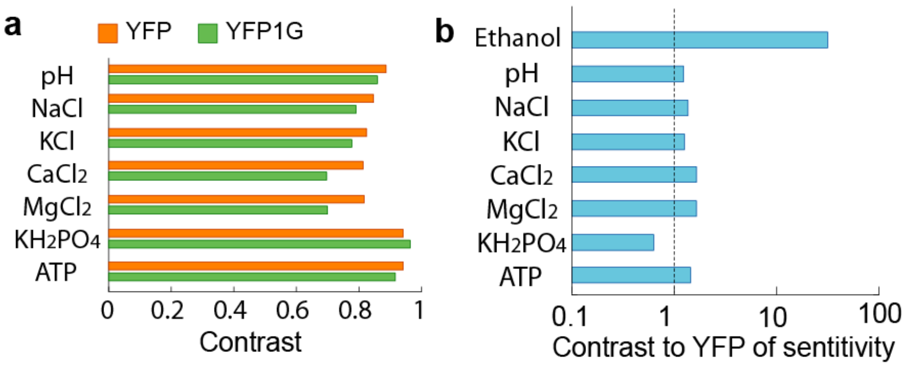

Figure S2. Dependencies of YFP and YFP1G fluorescence on various ions.

(**a**) Dependencies of fluorescence intensities of YFP (*orange*) and YFP1G (*green*) on various ion species. The pH dependence was defined as the ratio of the fluorescence intensities at pH 8.0 and 7.0. Other dependencies were defined as the ratio of the fluorescence intensities in 100 mM HEPES (pH 7.4) only and in 100 mM HEPES with 200 mM NaCl, 200 mM KCl, 200 mM $CaCl_2$, 200 mM $MgCl_2$, 200 mM KH_2PO_4, or 4 mM ATP, respectively. (**b**) Comparison of each dependency by taking the ratio of the changes for YFP1G and YFP. Ethanol values were obtained from Fig. S1.

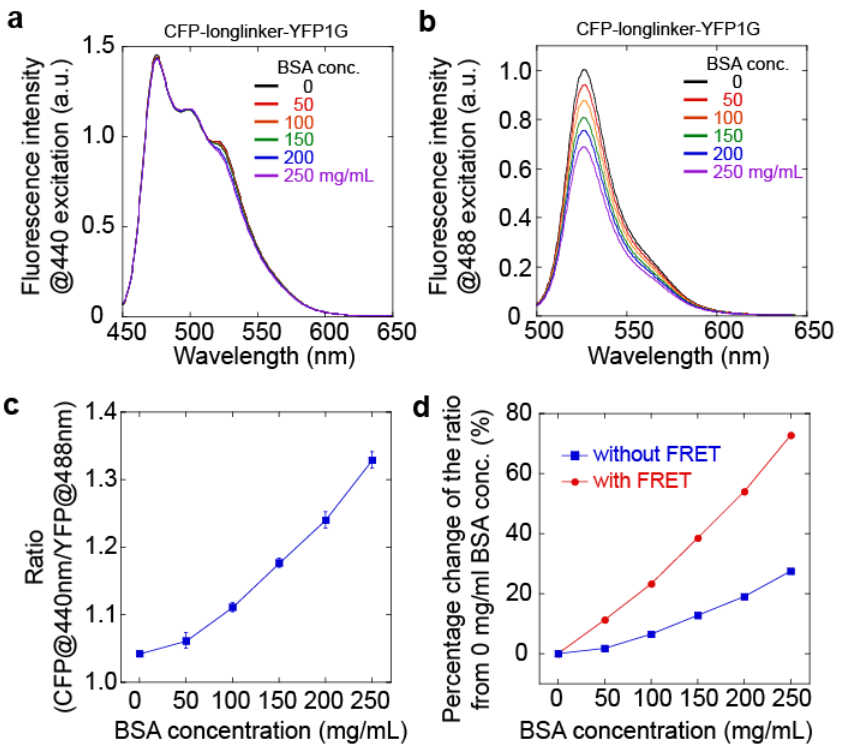

Figure S3. BSA concentration dependences of CFP-YFP1G without FRET.

(**a, b**) Fluorescence spectra of CFP-YFP1G linked with a long linker, GGSGGT×6**,** at 0–250 mg/mL BSA (*black*, 0 mg/mL; *red*, 50 mg/mL; *orange*, 100 mg/mL; *green*, 150 mg/mL; *blue*, 200 mg/mL; *purple*, 250 mg/mL). Excitation wavelengths were 440 nm (**a**) and 488 nm (**b**). Traces represent the average of four individual trials. (**c**) Relationship between BSA concentration and the intensity ratio between 460–500 nm (**a**) and 520–560 nm (**b**). (**d**) Change of the intensity ratio from 0 mg/mL BSA without (*blue*) and with (*red*) FRET calculated from **c** in this figure and Fig. 2c, respectively. All plots represent the average of four trials.

For CFP-longliker-YFP1G construction, we designed the sense primers containing the sequence encoding the long-linker (GGSGGT×6) and C-terminal sequence of CFP containing the BspEI site and reverse primers containing the BspEI site. The primers were annealed and ligated into a pAL7 vector encoding GimRET between the BspEI/BspEI sites and then transformed into Rosseta2 (DE3).

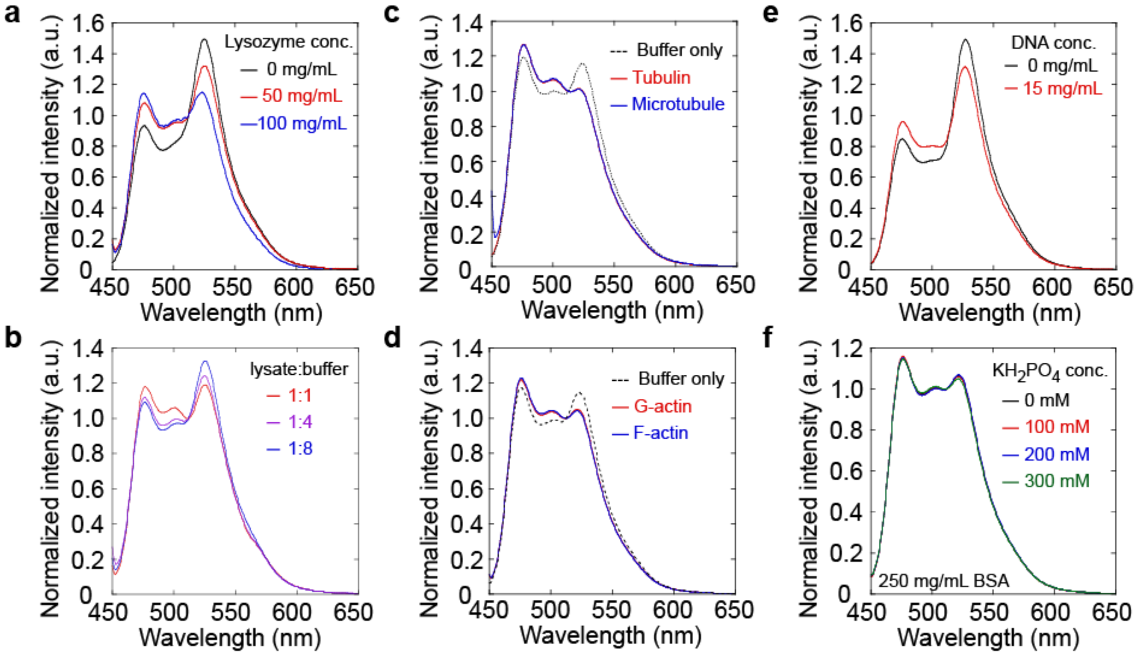

Figure S4. Dependences of GimRET fluorescence on various factors.

(**a**) Fluorescence spectra of GimRET at 0 mg/mL (*black*), 50 mg/mL (*red*), and 150 mg/mL lysozyme (*blue*). (**b**) Fluorescence spectra of GimRET in *E. coli* lysate. 1:1 (*red*), 1:4 (*purple*), and 1:8 (*blue*) dilutions of *E. coli*. (**c**) Fluorescence spectra of GimRET in the absence (*black*) or presence of 10 mg/mL tubulin (*red*) or microtubules (*blue*). (**d**) Fluorescence spectra of GimRET in the absence (*black*) or presence of 9.7 mg/mL actin monomer (G-actin, *red*) or actin filament (F-actin, *blue*). (**e**) Fluorescence spectra of GimRET in 0 mg/mL (*black*) and 15 mg/mL (*red*) DNA solutions. (**f**) Fluorescence spectra of GimRET at 0 mM (*black*), 100 mM (*red*), 200 mM (*blue*), and 300 mM (*green*) KH_2PO_4. All spectra were obtained at a 440 nm excitation wavelength, and represent the average of four trials.

Lysozyme chloride was dissolved into 100 mM HEPES (pH 7.4), and then the solution was dialyzed to remove the chloride ion. Actin was purified from rabbit skeletal muscle (1). To make actin filaments, we added 200 mM KCl and 10 µM phalloidin into the buffer, 2 mM HEPES (pH 7.8), 0.2 mM ATP, 0.1 mM $CaCl_2$, and 1 mM 2-mercaptoethanol. After 24 h, the actin filaments were collected by centrifuging to remove the chloride ions. Tubulin was purified from bovine brain (2). To make microtubule, we increased the temperature of the solution to 37 °C for 15 min, and then added 1 mM taxol into the buffer, 100 mM PIPES (pH 6.9), 1 mM $MgCl_2$, 1 mM EGTA, 1 mM GTP. To obtain the *E. coli* lysate, the cell pellet was mixed with 100 mM HEPES-NaOH (pH 7.4) until the weight of the wet pellet and buffer was the same, and sonicated for 30 s 20 times, resulting in *E. coli* solution (lysate:buffer = 1:1). The resultant protein concentration was 160 mg/mL. The *E. coli* solution was diluted in 100 mM HEPES-NaOH (pH 7.4) at a lysate:buffer ratio of 1:4 and 1:8. GimRET was diluted to 0.1 mg/mL in each *E. coli* lysate solution.

1 Spudich, J. A., & Watt, S. *J Biol Chem.* **246**, 4866–4871 (1971)

2 Murofushi, H., et al. *J Cell Biol.* **103**, 1911–1919 (1986)

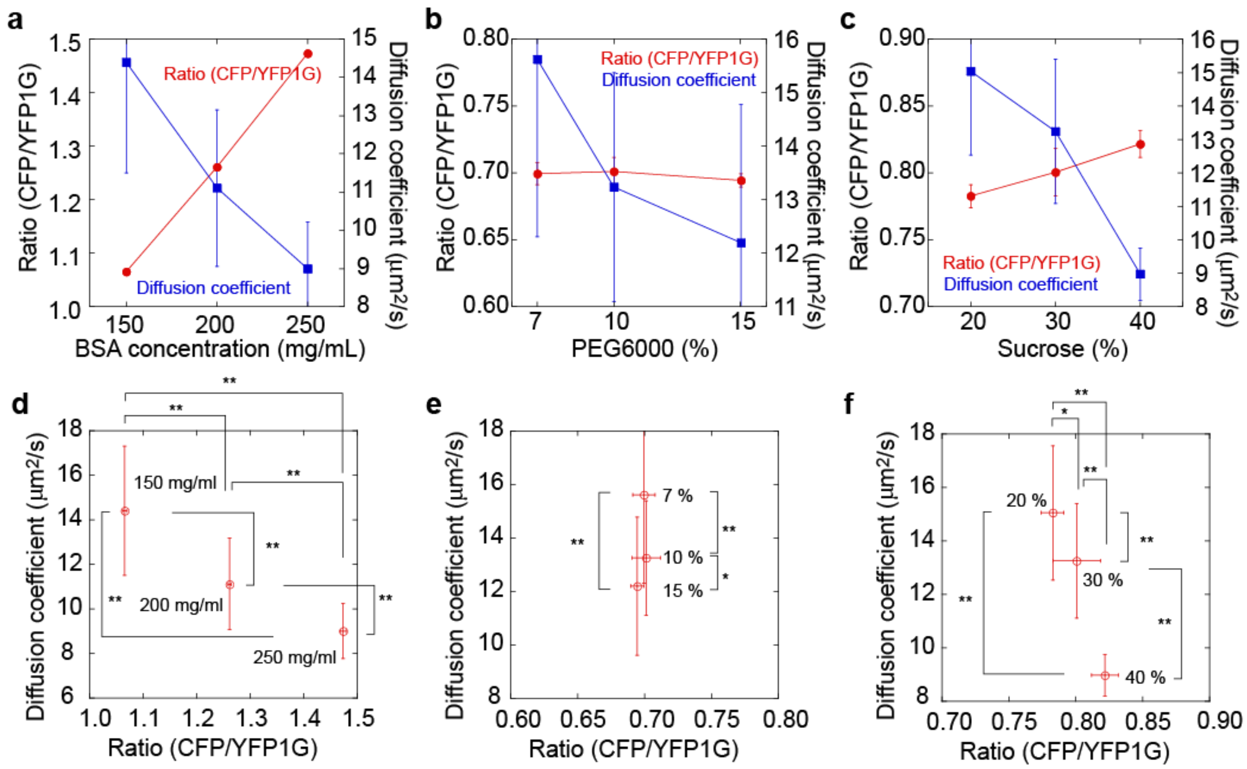

Figure S5. Relationship between intensity ratio and diffusion coefficient obtained by MP-FRAP in different solutions.

(**a–c**) Dependence of GimRET ratio and diffusion coefficient on various solution conditions. GimRET was diluted to 1 µg/mL in 150 mg/mL, 200 mg/mL, or 250 mg/mL BSA (**a**), 7.5%, 10%, or 15% (*v/v*) PEG6000 (**b**), and 20%, 30%, or 40% (*v/v*) sucrose (**c**). BSA, PEG6000, and sucrose were dissolved in 100 mM HEPES-NaOH (pH 7.4). *Red*, intensity ratio of CFP and YFP1G; *blue*, corresponding diffusion coefficients obtained by FRAP. Plots represent the average of 12 trials. Error bars, standard deviation. (**d–f**) Correlations of the diffusion coefficient obtained by FRAP and the intensity ratio of GimRET in BSA (**d**), PEG6000 (**e**), and sucrose (**f**). Data values are from **a**, **b**, and **c**, respectively. Error bars, standard deviation. Single and double asterisks correspond to P value is respectively < 0.05, and <0.01 in two sample t-test.

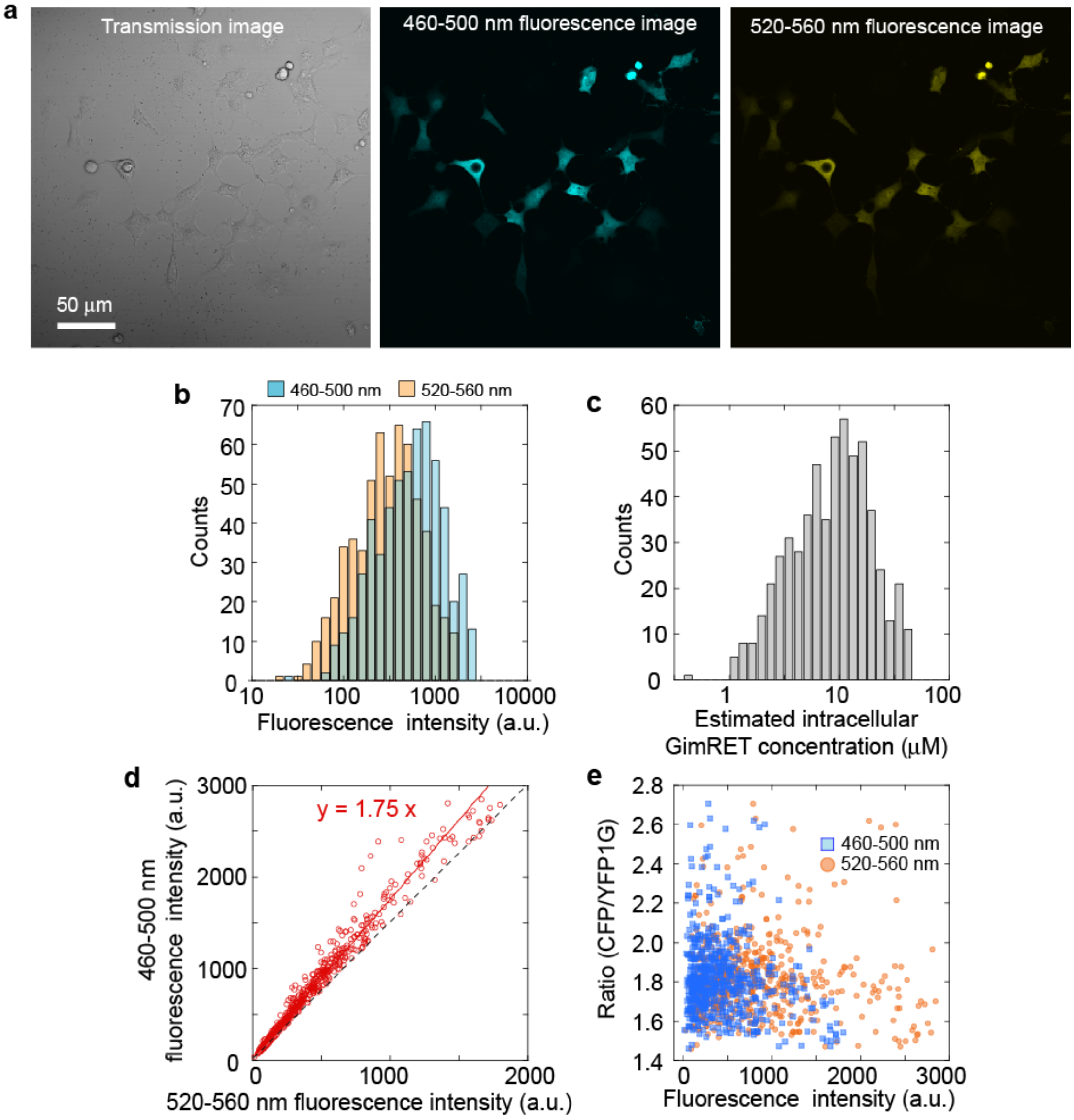

Figure S6. Relationship between the fluorescence and the intensity ratio of GimRET in Cos7 cells.

(**a**) Transmission image (*left*), fluorescence image at 460–500 nm (*middle*), and fluorescence image of Cos7 cells transfected with GimRET at 520–560 nm (*right*) using a low numerical aperture lens (25×, NA = 1.05). (**b**) Histogram of the fluorescence intensity of CFP (*blue*) and YFP1G (*yellow*) in Cos7 cells expressing GimRET. (**c**) Estimated intracellular GimRET concentration in Cos7 cells. The concentration was estimated from the calibration curve shown in Fig. S7a. (**d**) Correlation between the fluorescence intensity of CFP (460–500 nm) and YFP1G (520–560 nm) of whole cells. The solid line indicates the fit to a linear function. The broken line indicates a 1:1 correlation. Each plot indicates an individual single cell. (**e**) Correlation between the fluorescence intensity of CFP (*blue*) or YFP1G (*orange*) and the intensity ratio of GimRET. Each plot indicates an individual single cell, N = 578 cells. The estimation of the concentration of the expressed GimRET in cells was followed with the previously established procedure (3).

3 Niswender, K. D., et al. *J Microsc.* 180, 109–116 (1995)

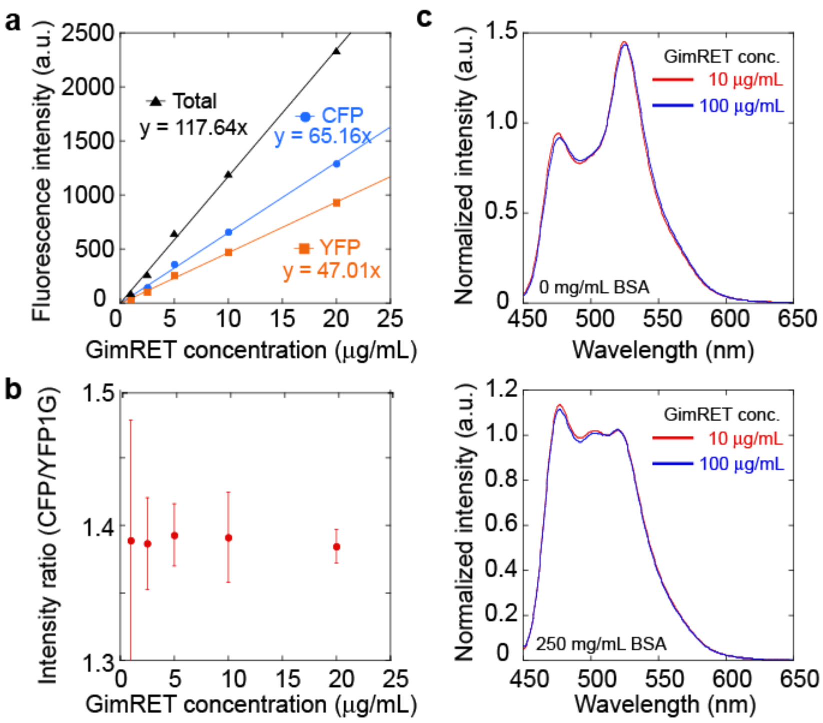

Figure S7. Effect of GimRET concentration on the calculation of the intensity ratio.

(**a**) Fluorescence intensity of CFP (*cyan*) and YFP1G (*orange*) of GimRET solutions at various concentrations measured under a fluorescent microscope. The total intensity was correlated depending on the intensity ratio (see Fig. 2c, *blue*). The purified GimRET at 1.0, 2.5, 5.0, 10, and 20 µg/mL was diluted into 250 mg/mL BSA (pH 7.4), and the fluorescence intensity of CFP (460–500 nm) and YFP (520 nm) were obtained on a photo detector using the fluorescent microscope. The standard deviation at the lower concentration (1.0 µg/mL) was larger than those at the higher concentration because of the low signal to noise ratio of the detection. (**b**) Relationship between the intensity ratio and GimRET concentration under the fluorescent microscope. The GimRET intensity ratios were calculated from **a**. (**c**) Fluorescent spectrum of 10 µg/mL (*red*) and 100 µg/mL (*blue*) GimRET in 0 mg/mL (*upper*) and 250 mg/mL (*lower*) BSA.

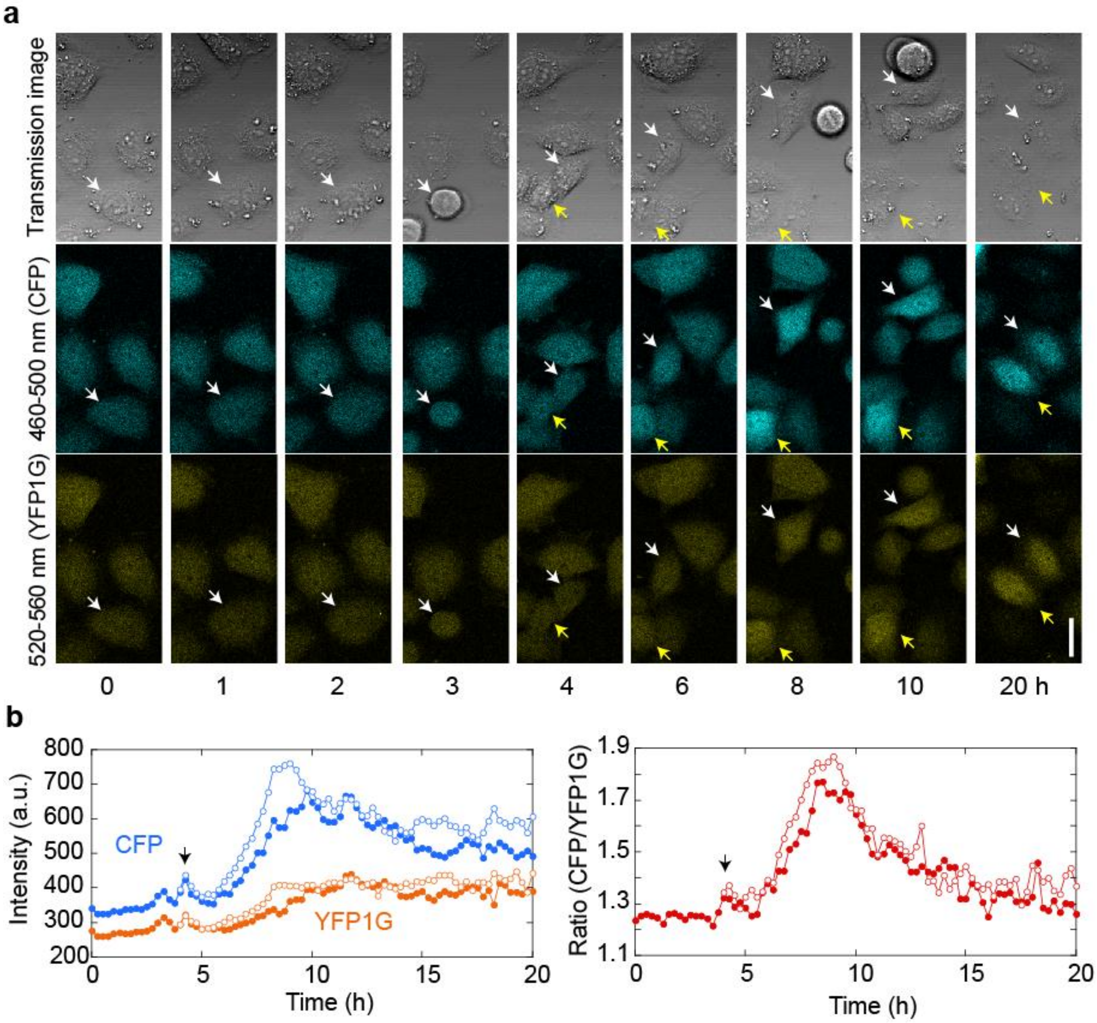

Figure S8. Intracellular measurement of GimRET fluorescence using a high numerical aperture objective lens. (**a**) Time-lapse transmission (*top*) and fluorescence images of 460–500 nm (CFP, *middle*) and 520–560 nm (YFP1G, *bottom*) of HeLa cells expressing GimRET using a high numerical aperture lens (60×, NA = 1.45). White arrows, mother cells. Yellow arrows, daughter cells. (**b**) Fluorescence intensity of CFP (*cyan*) and YFP1G (*orange*) and the intensity ratio of CFP and YFP1G (*red*) of the mother cells (*filled circles*) and daughter cells (*open circles*) in **a**. Black arrows indicate the moment of cell division.

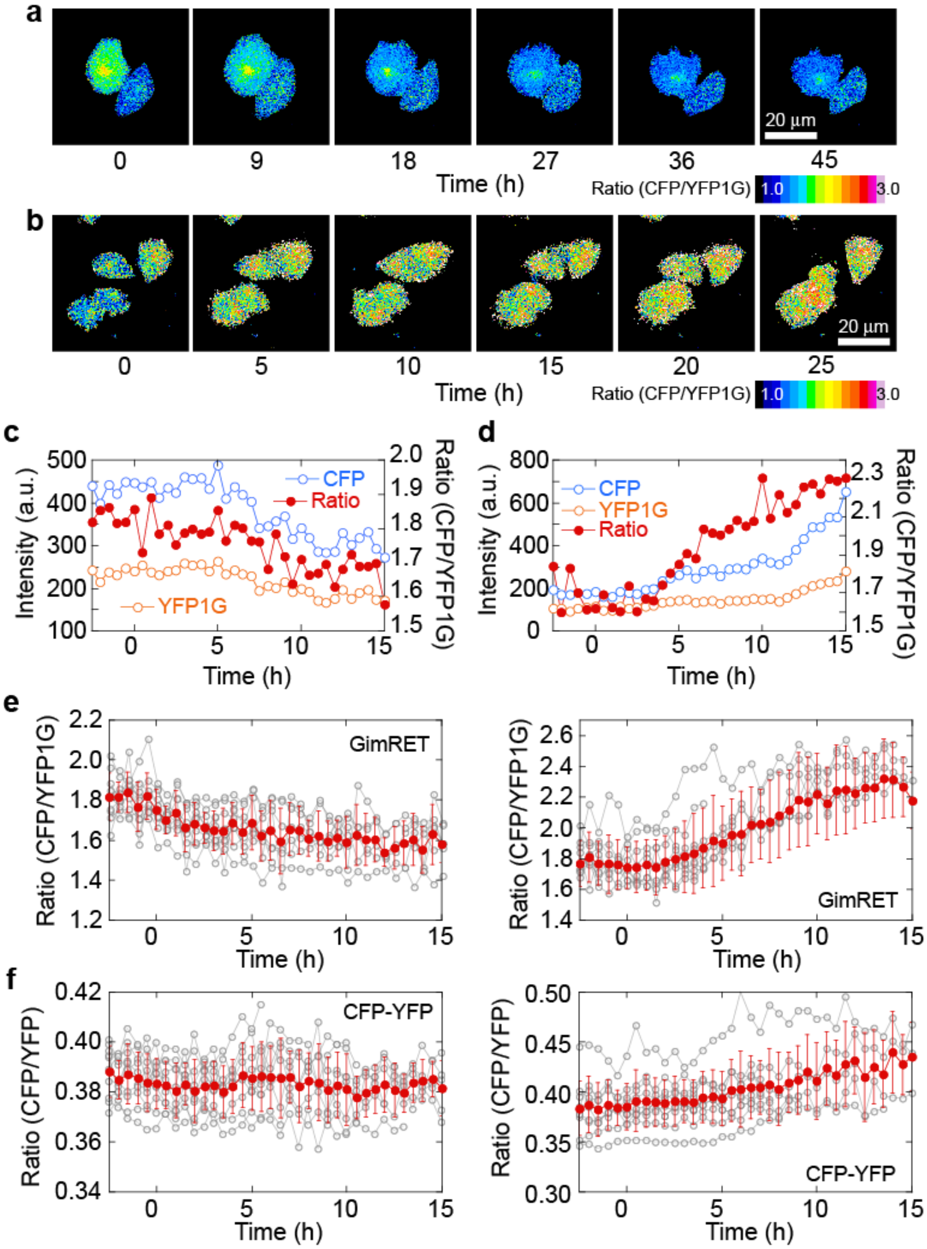

Figure S9. Effect of protein synthesis or degradation inhibition on GimRET measurement

(**a**, **b**) Time-lapse images of HeLa cells transfected with GimRET after addition of a translation inhibitor (cycloheximide, **a**) or proteasome inhibitor (MG132, **b**). Colored bars indicate the intensity ratio from 1.0 (*black*) to 3.0 (*white*). During image acquisition, 1 mM cycloheximide or 0.25 µM MG132 was added into the medium just before time 0. (**c**, **d**) Time course of the intensity of CFP (*cyan*) and YFP1G (*orange*) and their ratio (*red*) after adding cycloheximide (**c**) or MG132 (**d**). (**e**, **f**) Ten typical traces (*grey*) and average traces (*red*) of the intensity ratio of cells transfected with GimRET (**e**) or CFP-YFP (**f**) after adding cycloheximide (*left*) or MG132 (*right*). Error bars, standard deviations. The method is included in the legend of Fig. S11.

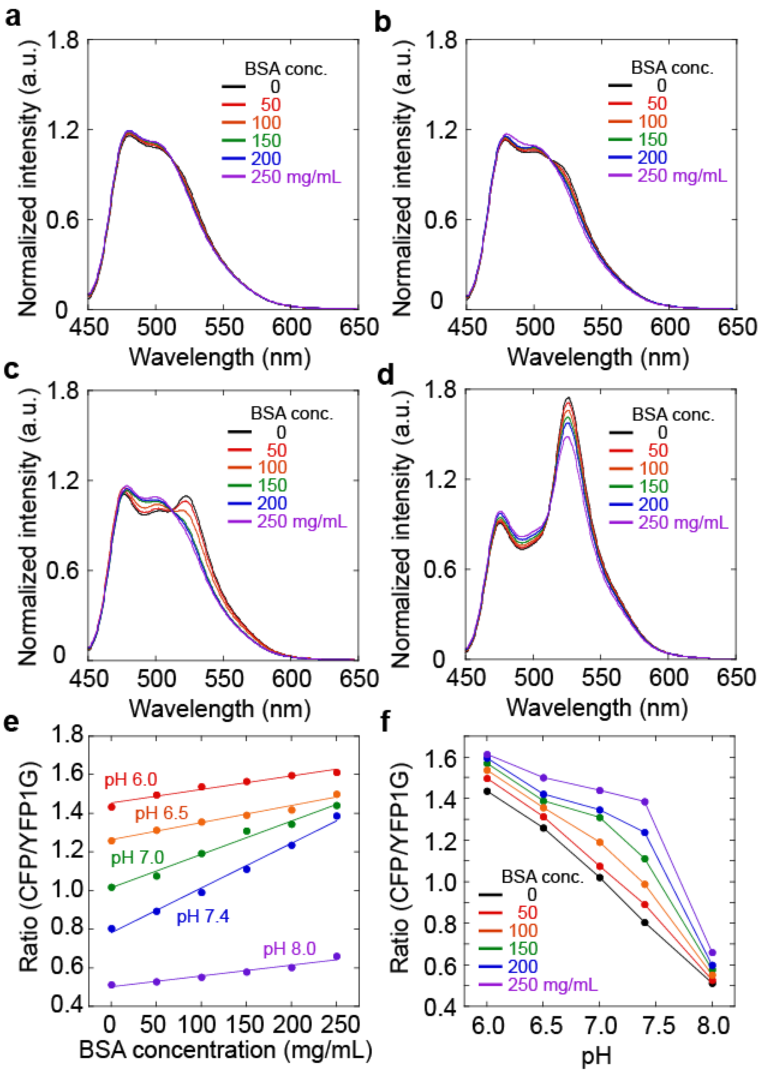

Figure S10. BSA concentration dependence of GimRET at various pHs.

(**a–e**) Fluorescence spectra of GimRET at 0–250 mg/mL BSA (*black*, 0 mg/mL; *red*, 50 mg/mL; *orange*, 100 mg/mL; *green*, 150 mg/mL; *blue*, 200 mg/mL; *purple*, 250 mg/ml) in 100 mM MES pH 6.0 (**a**), 100 mM MES pH 6.5 (**b**), 100 mM HEPES pH 7.0 (**c**), and 100 mM HEPES pH 8.5 (**d**). The excitation wavelength was 440 nm. (**e**) Relationship between BSA concentration and the ratio of intensity between 460–500 nm and 520–560 nm at various pHs (*red*, pH 6.0; *orange*, pH 6.5; *green*, pH 7.0; *blue*, pH 7.4; *purple*, pH 8.0). (**f**) Relationship between the ratio of intensity and the pH at 0–250 mg/mL BSA (*black*, 0 mg/mL; *red*, 50 mg/mL; *orange*, 100 mg/mL; *green*, 150 mg/mL; *blue*, 200 mg/mL; *purple*, 250 mg/mL). Traces or plots represent the average of four individual trials. The data for pH 7.4 is shown in Fig. 2b.

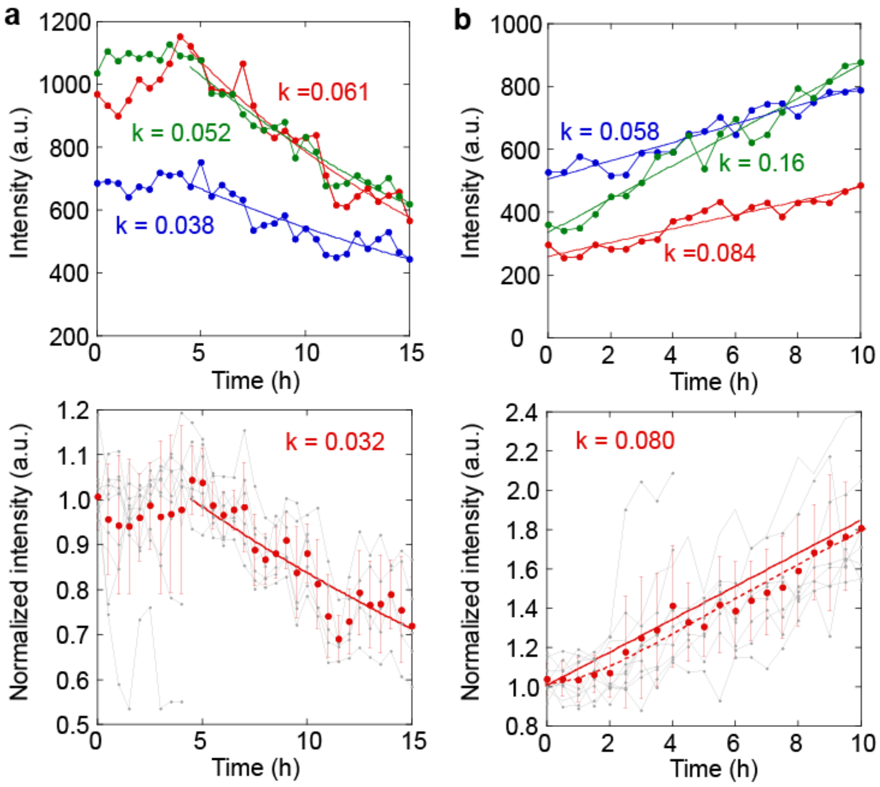

Figure S11 Synthesis and degradation of GimRET in HeLa cells.

(**a**) Typical time courses (*upper*) and an average trace (*lower*) of the total intensity of CFP and YFP1G after adding a translation inhibitor (cycloheximide). Grey plots in the lower panel show traces for ten trials. The solid lines are fits to single exponential curves. The values shown are the estimated degradation rates. (**b**) Typical time courses (*upper*) and an average trace (*lower*) of the total intensity of YFP1G and CFP after adding a proteasome inhibitor (MG132). Grey plots in the lower panel show traces for ten trials. The solid lines are fits to linear curves, $f(t) = a \cdot k \cdot t + a$. The values shown are the estimated synthesis rate. The broken line is the fit to a model function considering maturation, $f(t) = 1 + k_1 \cdot t \cdot \{1 – \exp(-k_2 \cdot t)\}$, where k_1 is the synthesis rate and k_2 is the maturation rate (k_1 = 0.075 h^{-1} and k_2 = 0.40 h^{-1}). Data are from Fig. S9.

HeLa cells expressed GimRET or CFP-YFP were observed under the fluorescent microscope under 5% CO_2 at 37°C for 50 h. The time interval was 30 min. In the case of protein synthesis inhibition or protein degradation inhibition, we changed medium with 1 mM translation inhibitor (cycloheximide, WAKO, JP) or 0.25 µM proteasome inhibitor (MG132, WAKO, JP) on the fluorescent microscope 2.5 h after the start of observation. The observation was performed by using 25× and 60× objective lens. The fluorescent images were averaged 5 times. The analysis of the data was performed using Image-J.

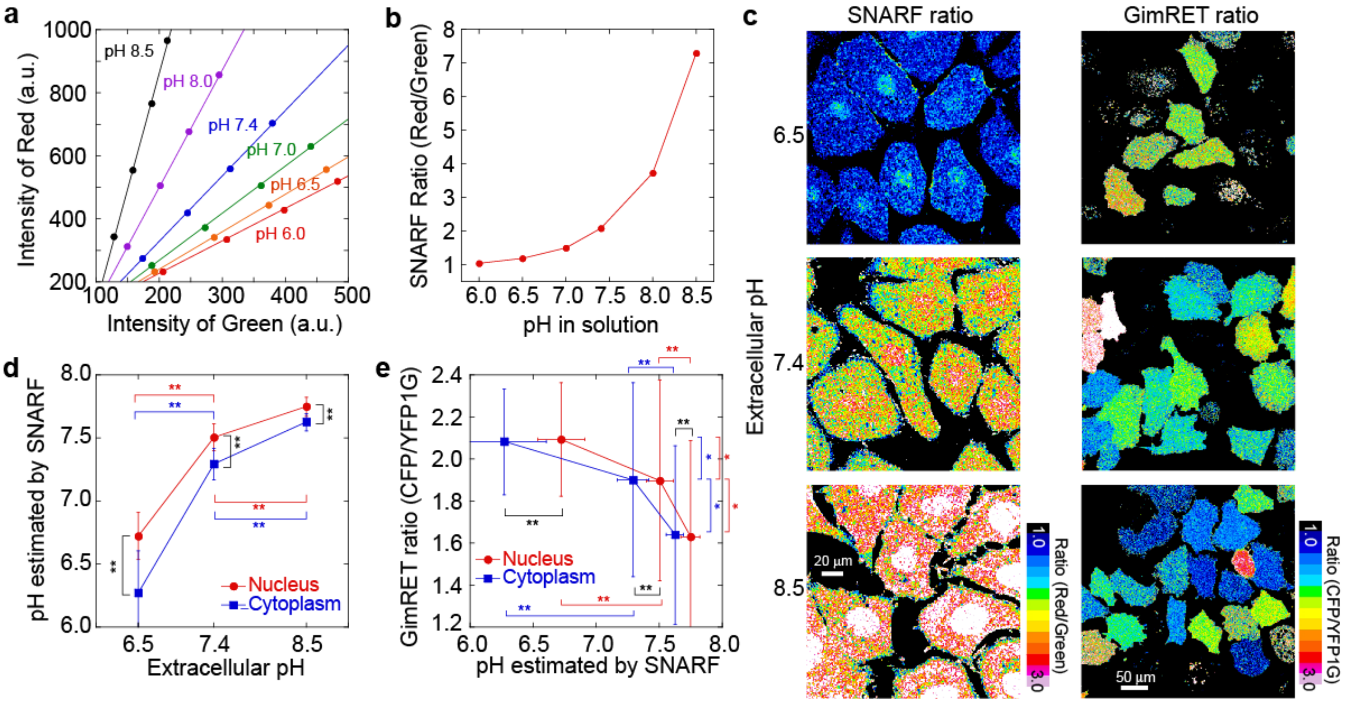

Figure S12. SNARF-1 and GimRET observation during intracellular pH change.

(**a**) Correlation curves of fluorescence intensity for 10 µM SNARF-1 in red (640–700 nm) and green (500–560 nm) channels at various exaction powers in pH 6.0–8.5 (*red*, pH 6.0; *orange*, pH 6.5; *green*, pH 7.0; *blue*, pH 7.4; *purple*, pH 8.0; *black*, pH 8.5). Each best-fit line equals each ratio value at the specified pH without considering the background intensity. (**b**) Calibration curve of the SNARF-1 ratio (red channel/green channel) and the pH in solution. (**c**) Ratiometric images of HeLa cells labelled with SNARF-1 (*left*) and expressing GimRET (*right*) at extracellular pH 6.5 (*top*), 7.4 (*middle*), and 8.5 (*bottom*). Coloured bars indicate the intensity ratio from 1.0 (*black*) to 3.0 (*white*). (**d**) The intracellular pH estimated by the SNARF-1 ratio in the nucleus (*red*) and cytoplasm (*blue*) at extracellular pH 6.5 (N = 36), 7.4 (N = 77), and 8.5 (N = 48). (**e**) Relationship between the GimRET ratio and intracellular pH estimated by SNARF-1 in the nucleus (*red*) and cytoplasm (*blue*) at extracellular pH 6.5 (N = 29 for GimRET and 36 for SNARF), 7.4 (N = 76 and 77), and 8.5 (N = 48 and 48). Error bars, standard deviations. Single and double asterisks correspond to P value is respectively < 0.01, and <0.001 in two sample t-test.

Cells expressing GimRET, CFP-YFP or stained with SNARF were incubated in 20 mM PBS (pH 7.4) for 10 min before observation, and the cells were observed under the fluorescent microscope. After the observation, we changed the medium from PBS at pH 7.4 to PBS at pH 8.5 or pH 6.5 on the microscope to change the extracellular pH. After 30 min, we observed the same cells. Because intracellular pH was stable, we observed the cells 30 min after change of the medium. The observation was performed by using 60× objective lens. The fluorescent images were averaged 5 times. The data analysis was performed using homemade software.

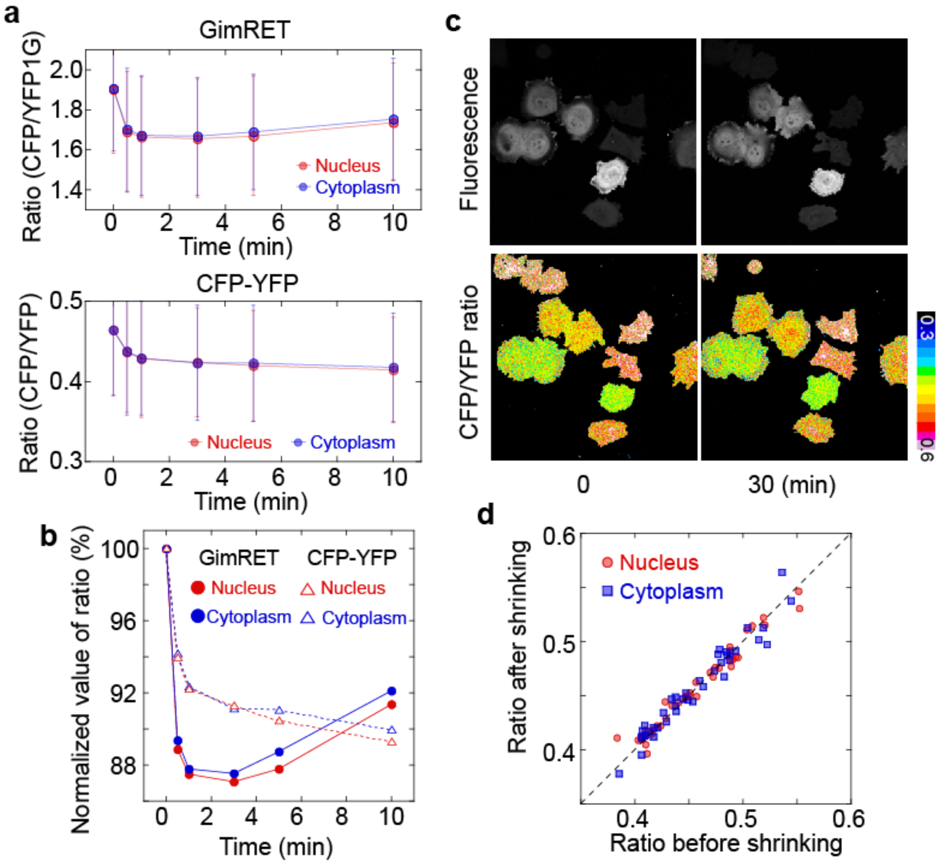

Figure S13 Control experiments in forcibly swelling and shrinking cells using CFP-YFP.

(**a**) Average traces of the intensity ratio of the nucleus (*red*) and cytoplasm (*blue*) in HeLa cells transfected with GimRET (*upper*, N = 34) or CFP-YFP (*lower*, N = 17) after adding hypo-osmotic medium. Error bars, standard deviations. (**b**) Normalized values of the intensity ratio of the nucleus (*red*) and cytoplasm (*blue*) in cells transfected with GimRET (*filled circles*, N = 34) or CFP-YFP (*open triangles*, N = 17). (**c**) Fluorescence images of the total fluorescent intensity (*upper*) and the ratio between 460–500 nm and 520–560 nm (*lower*) of cells expressing GimRET before (*left*) and 30 min after (*right*) adding 10 μM nocodazole. (**d**) Correlation between the intensity ratios before and 30 min after adding nocodazole in the nucleus (*red*) and cytoplasm (*blue*). Each plot indicates a single cell, N = 40. The broken line indicates a 1:1 correlation.

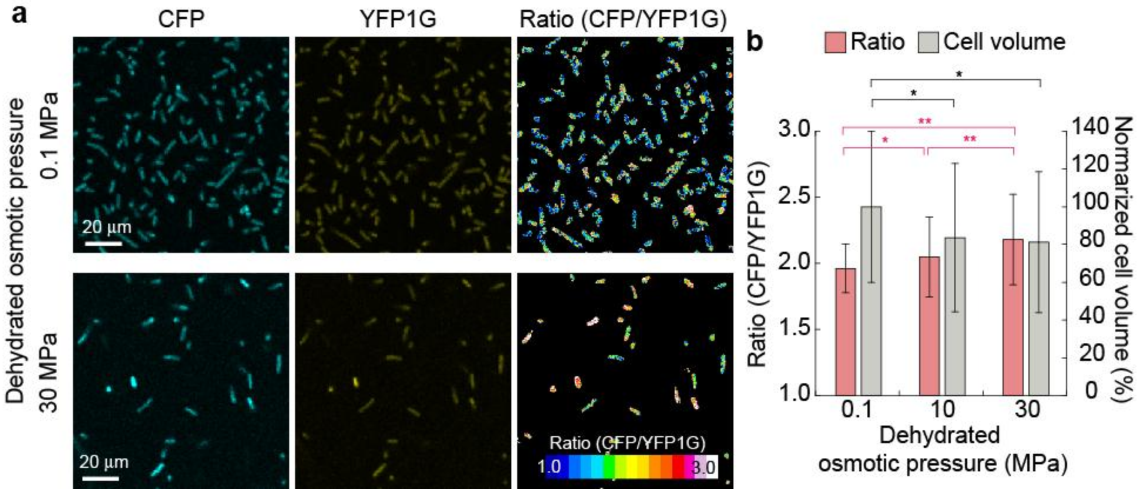

Figure S14. Osmotic shock assay of *E. coli* using GimRET.

(**a**) Fluorescence images of CFP- (*left*) and YFP1G-channel (*middle*) and ratiometric image (*right*) of *E. coli* expressing GimRET at 0.1 MPa (*upper*) and 30 MPa (*lower*). Colour bar indicates the intensity ratio from 1.0 (*black*) to 3.0 (*white*). (**b**) Bar graphs of the intensity ratio of GimRET (*red*) and the cell volume (*grey*) at 0.1 MPa (N = 416), 10 MPa (N = 233), and 30 MPa (N = 212) dehydrated osmotic pressure. Error bars, standard deviations. Single and double asterisks correspond to P value is respectively < 0.01, and <0.001 in two sample t-test.

The procedure of the osmotic shock assay for *E. coli* by glycerol was performed following the previous report (4). The dehydrated osmotic pressure was calculated using the Norrish equation (5). Glycerol diluted in 27% (10 MPa), and 50% (30 MPa) with 100 mM HEPES buffer (pH 7.4). *E. coli* expressing GimRET was mixed in 100 mM HEPES buffer (pH7.4) or each glycerol diluted solution, and placed on the poly-L- lysine (Sigma-Aldrich, MO) -coated coverslips (Matsunami, JP). 10 min after, the *E. coli* were observed under fluorescent microscope. The observation was performed by using 60× objective lens. The fluorescent images were averaged 5 times. The data analysis was performed using ImageJ. The calculation method of cell volume was previously described (6).

4 Mille, Y., Beney, L., & Gervais, P. *Biochim Biophys Acta.* 1567:41-48 (2002)

5 Norrish, R. S. *J. Food Technol.* 1: 25–39 (1966).

6 Cookson, N. A., Cookson, S. W., Tsimring, L. S. & Hasty, J. *Nucleic Acids Res.* 38, 2676-2681 (2010)

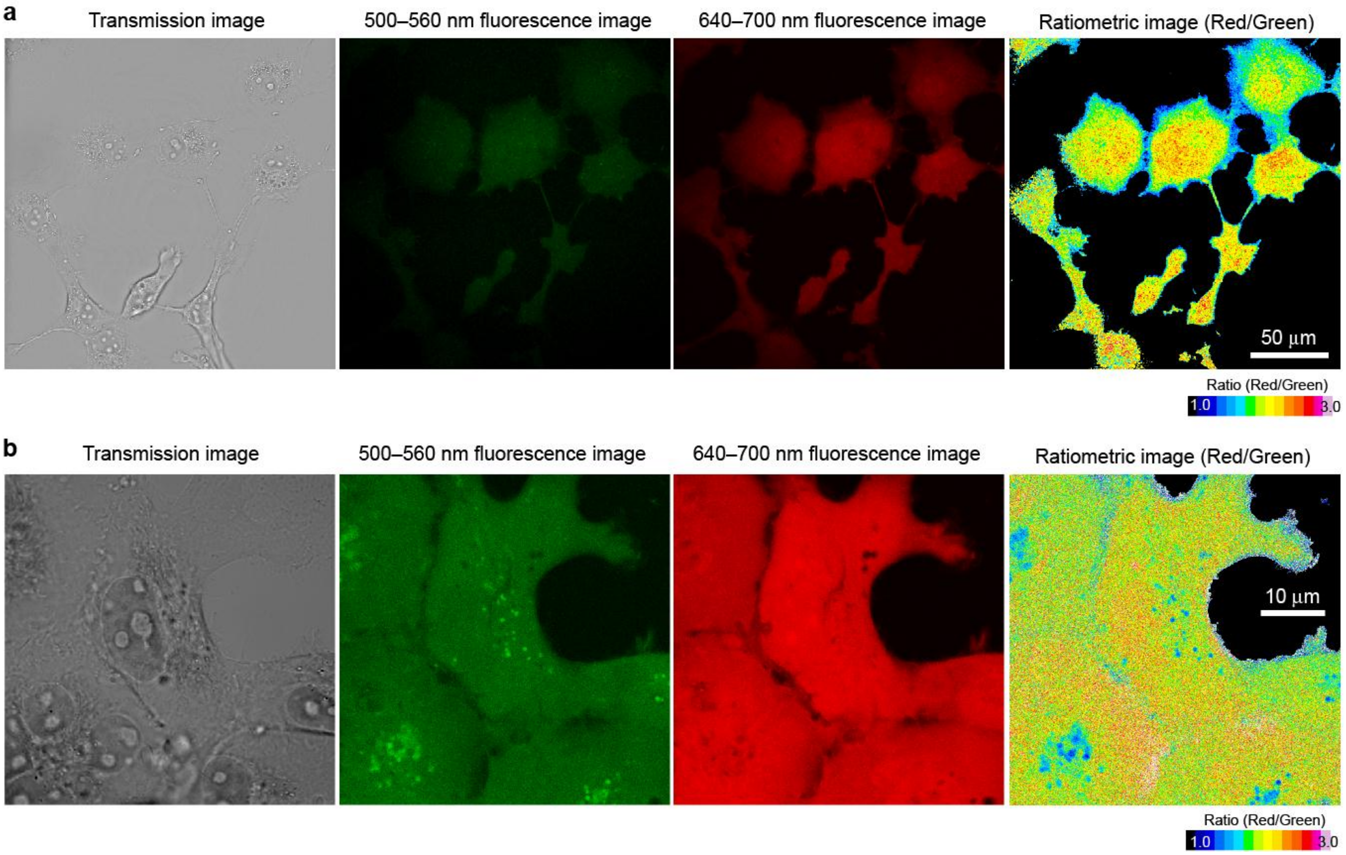

Figure S15. High-resolution observation of the intracellular pH of Cos7 cells by SNARF-1.

(**a**) Fluorescence images of Cos7 cells labelled with SNARF-1 acetoxymethyl ester. *Left*, transmission image; *middle left*, green channel (500–560 nm); *middle right*, red channel (640–700 nm); *right*, ratiometric image. The ratio was calculated by dividing the red channel by the green channel in each pixel. Colour bar indicates the ratio from 1.0 (*black*) to 3.0 (*white*). (**b**) High-resolution transmission image of Cos 7 cells labelled with SNARF-1. Panels are as in **a**. Colour bar indicates the ratio from 1.0 (*black*) to 3.0 (*white*).

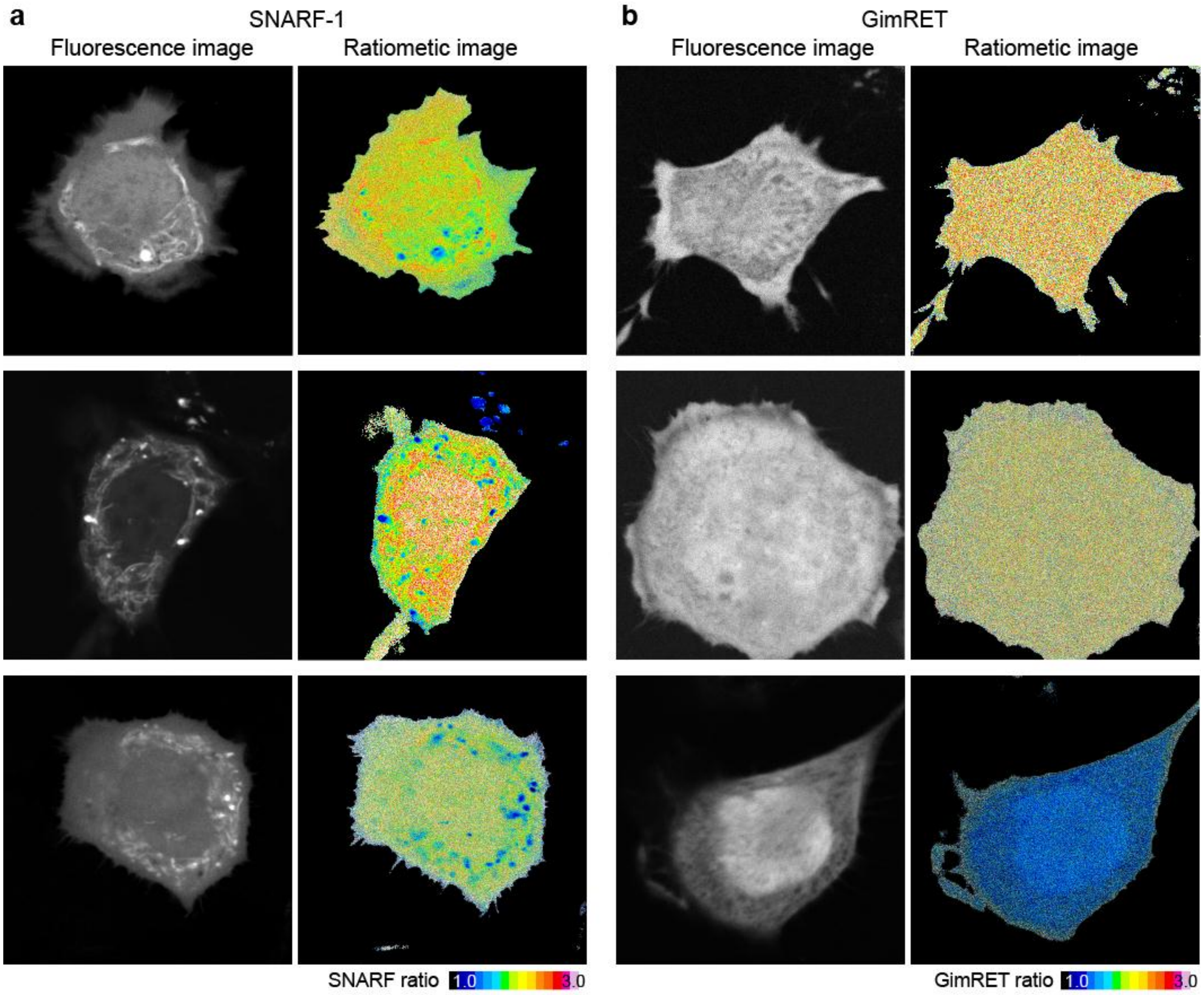

Figure S16. Comparison of ratiometric images of SNARF-1 and GimRET in Neuro 2A cells.

Images of the total fluorescent intensity (*left panels*) and the intensity ratio (*right panels*) of Neuro 2A cell labelled with SNARF-1 (**a**) or expressing GimRET (**b**). The ratio value for SNARF-1 is the ratio of 640–700 nm and 500–560 nm, and that of GimRET is the ratio of 460–500 nm and 520–560 nm. Colour bars indicate the ratio from 1.0 (*black*) to 3.0 (*white*).

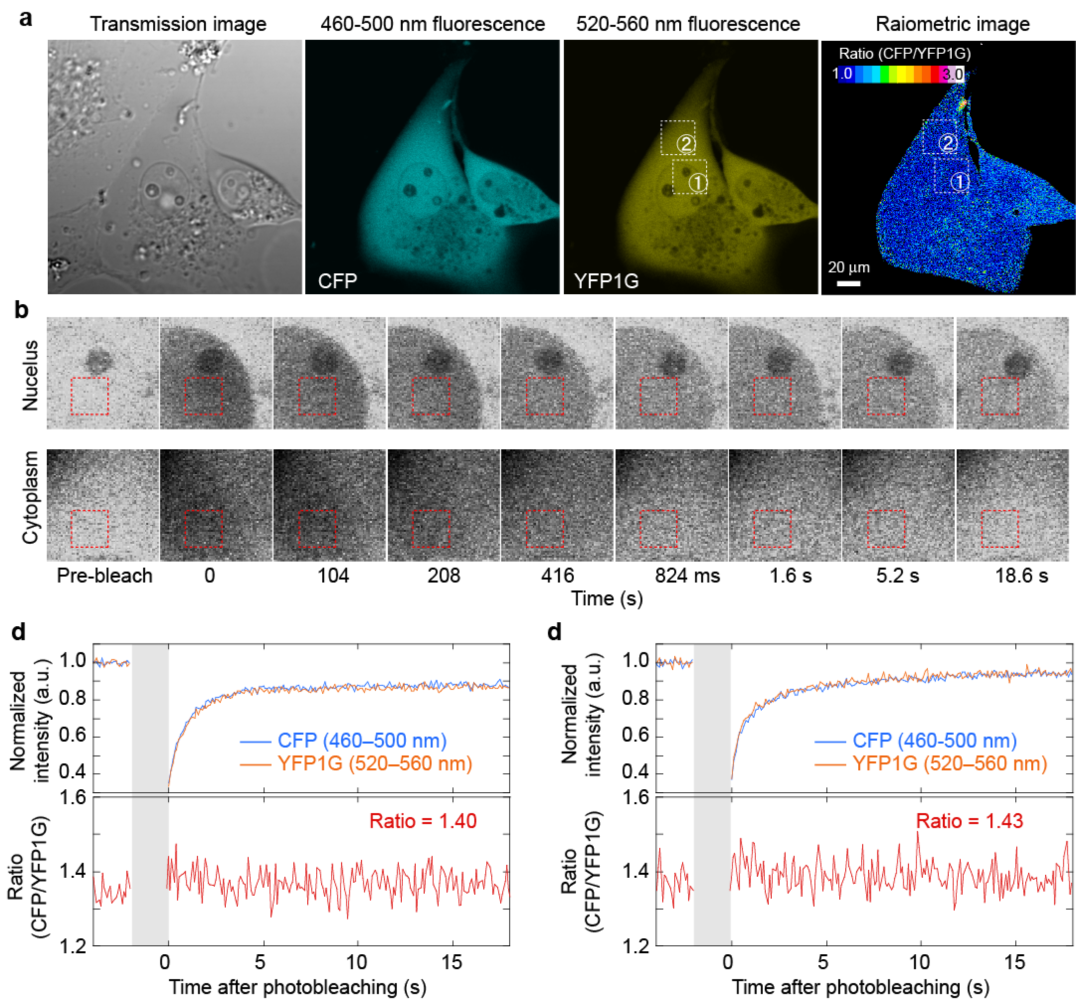

Figure S17. MP-FRAP assay of GimRET in a living cell.

(**a**) Transmission image (*left*), fluorescence images at 460–500 nm (*middle left*) and 520–560 nm (*middle right*), and ratiometric image (*right*). Colour bar indicates the ratio from 1.0 (*black*) to 3.0 (*white*). (**b**) Sequential images of FRAP experiment performed in the area indicated by the rectangles in **a** (*upper row*, #1; *lower row*, #2). (**c, d**) Photorecovery curves of the nucleus (**c**) and cytoplasm (**d**), which had almost the same ratio (~1.4). *Upper panels*, fluorescence intensity of CFP (*cyan*) and YFP1G channels (*orange*). *Lower*, ratio of CFP and YFP1G.

The observation area was limited to 10.56×0.56 μm^2 (64×64 pixels) to increase the temporal resolution to 0.01 s (exposure time, 0.008192 s). The photobleached area was 3×3 μm^2 (20×20 pixels). Photobleaching was done by illuminating the area for 2 s at maximum laser power after 3 s prebleaching observation. Photorecovery was observed for 27 s. We set the illumination power of the laser as weak as possible during photorecovery observation, because photobleaching caused a complex photorecovery curve. Total fluorescence intensity was maintained at 100% during the 30 s in the area that was not photobleached. The time course of fluorescent recovery in photobleached area was measured, and fitted to the following equation, I = 1-exp(-kt), where I is fluorescent intensity, t is time (s), k is recovery constant

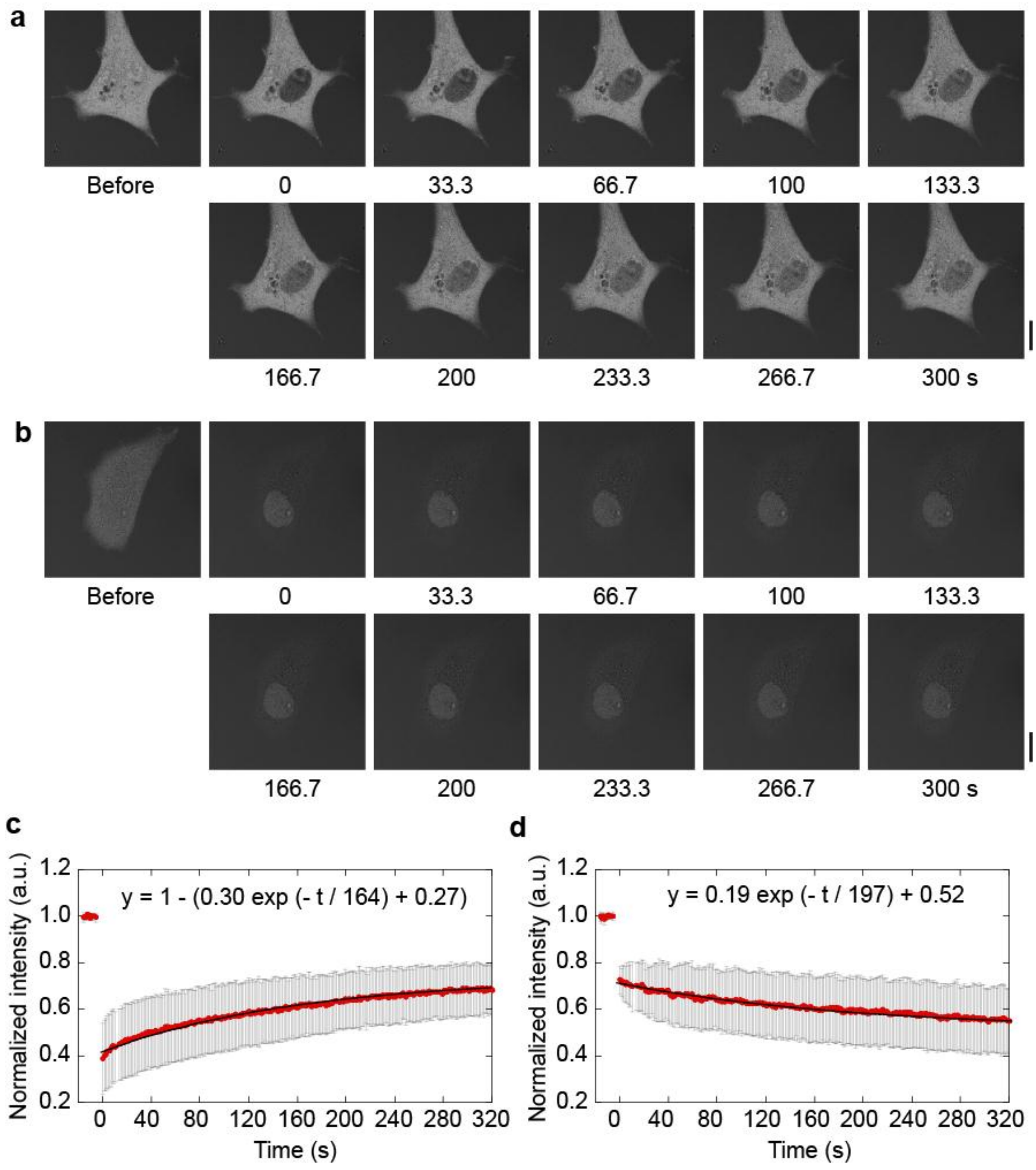

Figure S18. Measurement of the nuclear import/export rate of GimRET.

(**a, b**) Sequential images of FRAP inside (**a**) and outside (**b**) the nucleus. We photobleached the whole area of the nucleus (**a**) or the cytoplasm (**b**) then measured the fluorescence change. Scale bars, 20 µm. (**c, d**) Average traces of the fluorescence intensity of GimRET after photobleaching the inside (**c**) and outside (**d**) of the nucleus (N = 10). Error bars, standard deviations. The recovery (**c**, import) or decay (**d**, export) times were obtained by fits to a single exponential curve (import: 164 s, export: 197 s).

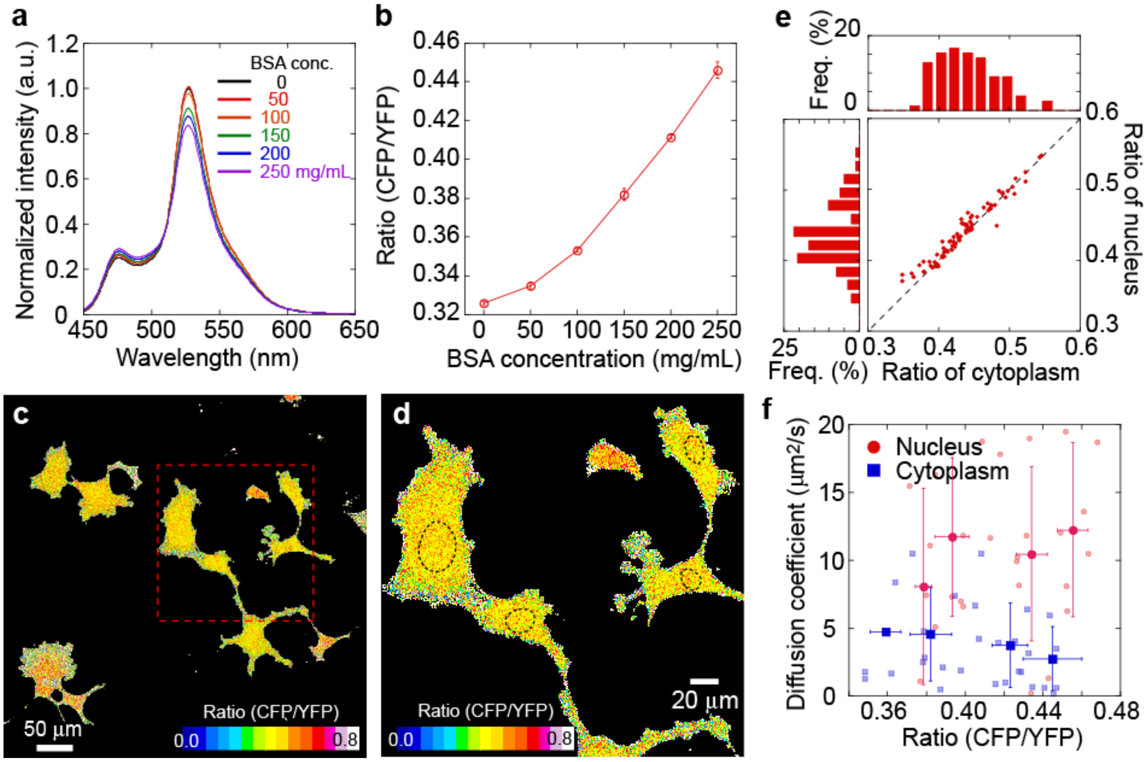

Figure S19. Control experiments in FRAP using CFP-YFP.

(**a**) Fluorescence spectra of CFP-YFP at 0–250 mg/mL BSA (*black*, 0 mg/mL; *red*, 50 mg/mL; *orange*, 100 mg/mL; *green*, 150 mg/mL; *blue*, 200 mg/mL; *purple*, 250 mg/mL). Traces represent the average of four trials. (**b**) Relationship between BSA concentration and the ratio of CFP and YFP. Plots represent the average of four trials. Error bars, standard deviation. (**c**) Low-magnification ratiometric image of Cos7 cells transfected with CFP-YFP. (**d**) Enlargement of the region indicated by the broken red rectangle in **c**. Black broken lines indicate the nucleus. Colour bar indicates the ratio from 0.0 (*black*) to 0.8 (*white*). (**e**) Relationship between the ratios from the cytoplasm and nucleus (*right lower*), and histograms of the ratios from the cytoplasm (*upper*) and nucleus (*left*). The broken line indicates a 1:1 correlation. N = 80 cells. (**f**) Relationship between the ratio and the diffusion coefficient obtained with MP-FRAP in the cytoplasm (*blue*) and nucleus (*red*). N = 31 cells. Error bars, standard deviations. Light-coloured symbols indicate individual cells.

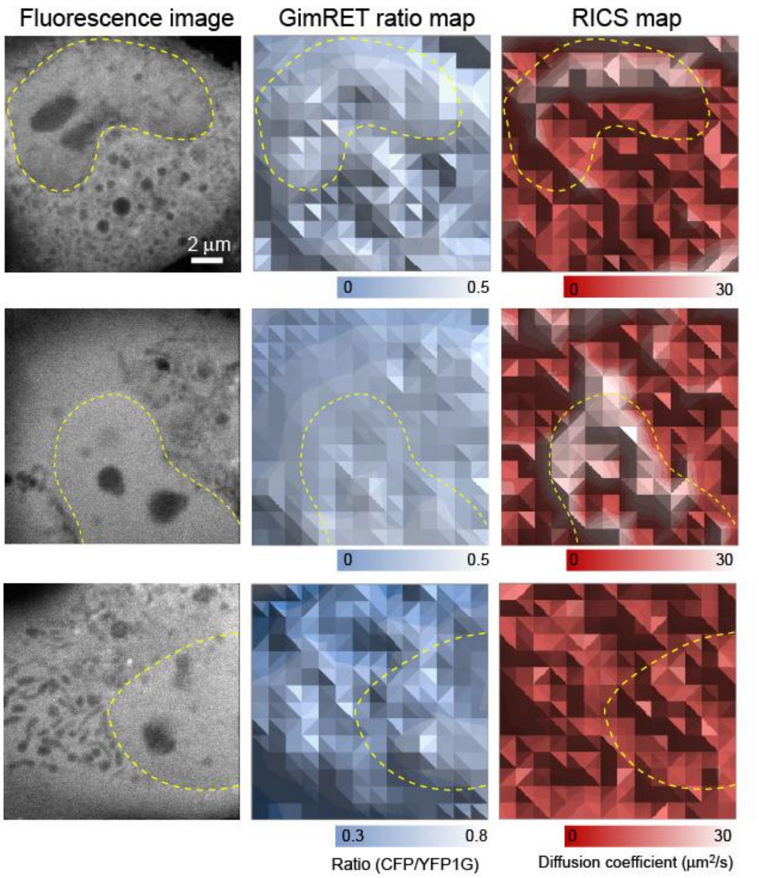

Figure S20. RICS diffusion mapping and GimRET ratio molecular crowding mapping

High-magnification fluorescent image (*left*), GimRET map (*middle*) and 15×15 RICS diffusion map (*right*). Colour bars indicate the ratio from 0 or 0.3 (*blue*) to 0.5 or 0.8 (*white*) and 0 (*red*) to 30 (*white*), respectively. Yellow broken lines indicate the boundary between the nucleus and the cytoplasm.

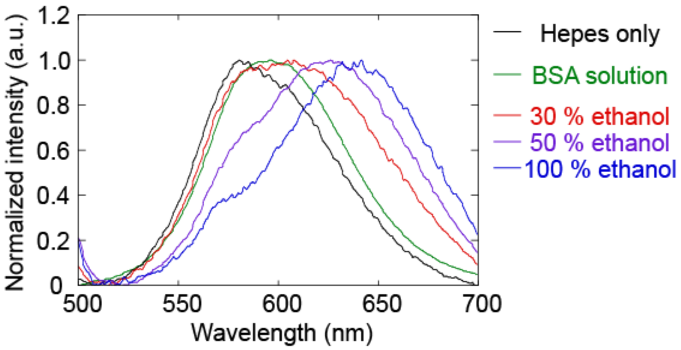

Figure S21. Fluorescence spectral shift of POLARIC in BSA and ethanol.

Colours indicate the solution conditions (*black*, HEPES; *green*, 50 mg/mL BSA; *red*, 30% ethanol; *magenta*, 50% ethanol; *blue*, 100% ethanol). Plots represent the average of four trials. POLARIC (GORYO Chemical imc.) was diluted to 10 μM in 100 mM HEPES-NaOH (pH 7.4), in 100 mM HEPES-NaOH (pH 7.4) with 250 mg/ml BSA, or in 30% (*v/v*), 50% (*v/v*), and 100% (*v/v*) ethanol solution, and the fluorescence spectrum was measured. The excitation wavelength was set to 488 nm, and the emission was scanned from 500–700 nm.

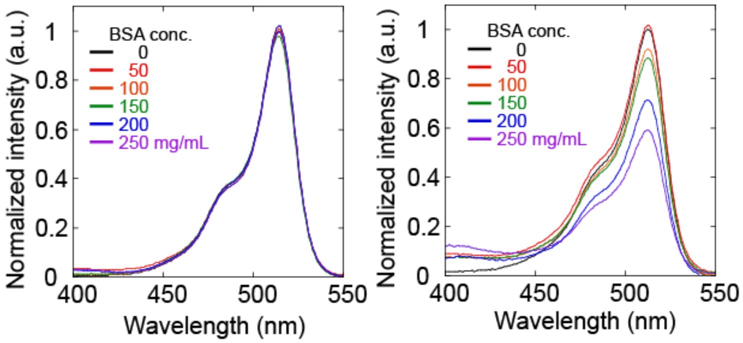

Figure S22. Absorbance spectra of YFP (*left*) and YFP1G (*right*) at 0–250 mg/mL BSA.

Colours indicate the BSA concentration (*black*, 0 mg/mL; *red*, 50 mg/mL; *orange*, 100 mg/mL; *green*, 150 mg/mL; *blue*, 200 mg/mL; *purple*, 250 mg/mL). BSA was dissolved in 100 mM HEPES-NaOH (pH 7.4). Plots represent the average of four trials. GimRET was diluted to 0.1 mg/mL in each BSA solution, and the absorbance was scanned from 400–550 nm (Shimadzu UV-Vis Spectrophotometer UV-1650PC). The intensity was normalized to 0 mg/mL BSA. The baseline spectrum of each BSA concentration was measured without GimRET and subtracted from each sample.

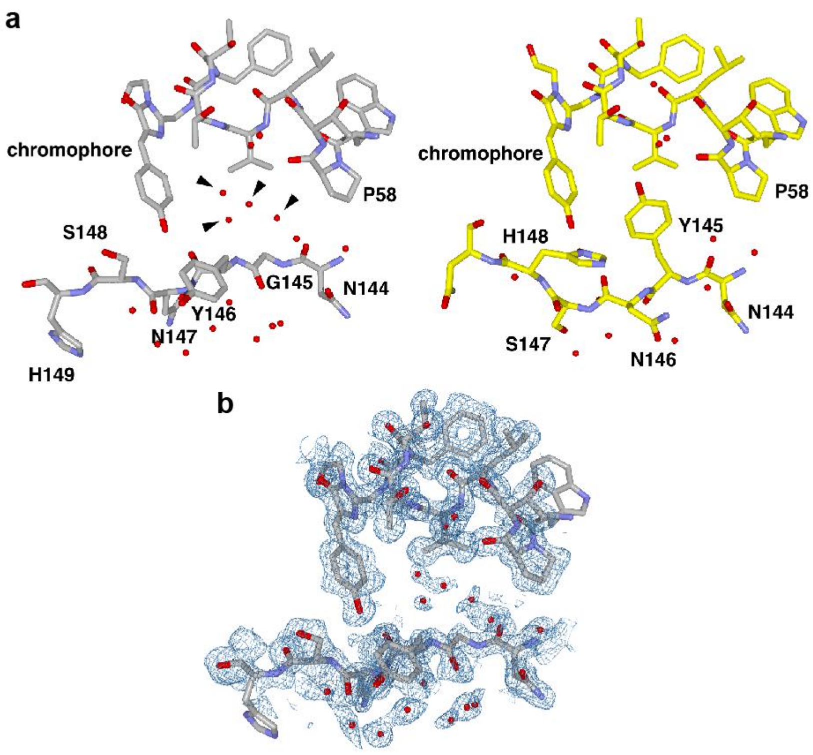

Figure S23. Crystal structure of the chromophore periphery of glycine-inserted YFP.

(**a**) Comparison of the chromophore structures of YFP-1G (*left*, PDB ID: 1yfp) and YFP (*righ*, PDBID: 3vgq). Water molecules are represented by red ball. The arrows indicate water molecules filling the space where Gly145 of YFP was located. Oxygen and nitrogen atoms are colored in red and blue, respectively. (**b**) Superimpose view of the YFP-1G structure shown with the 2Fo-Fc density map.

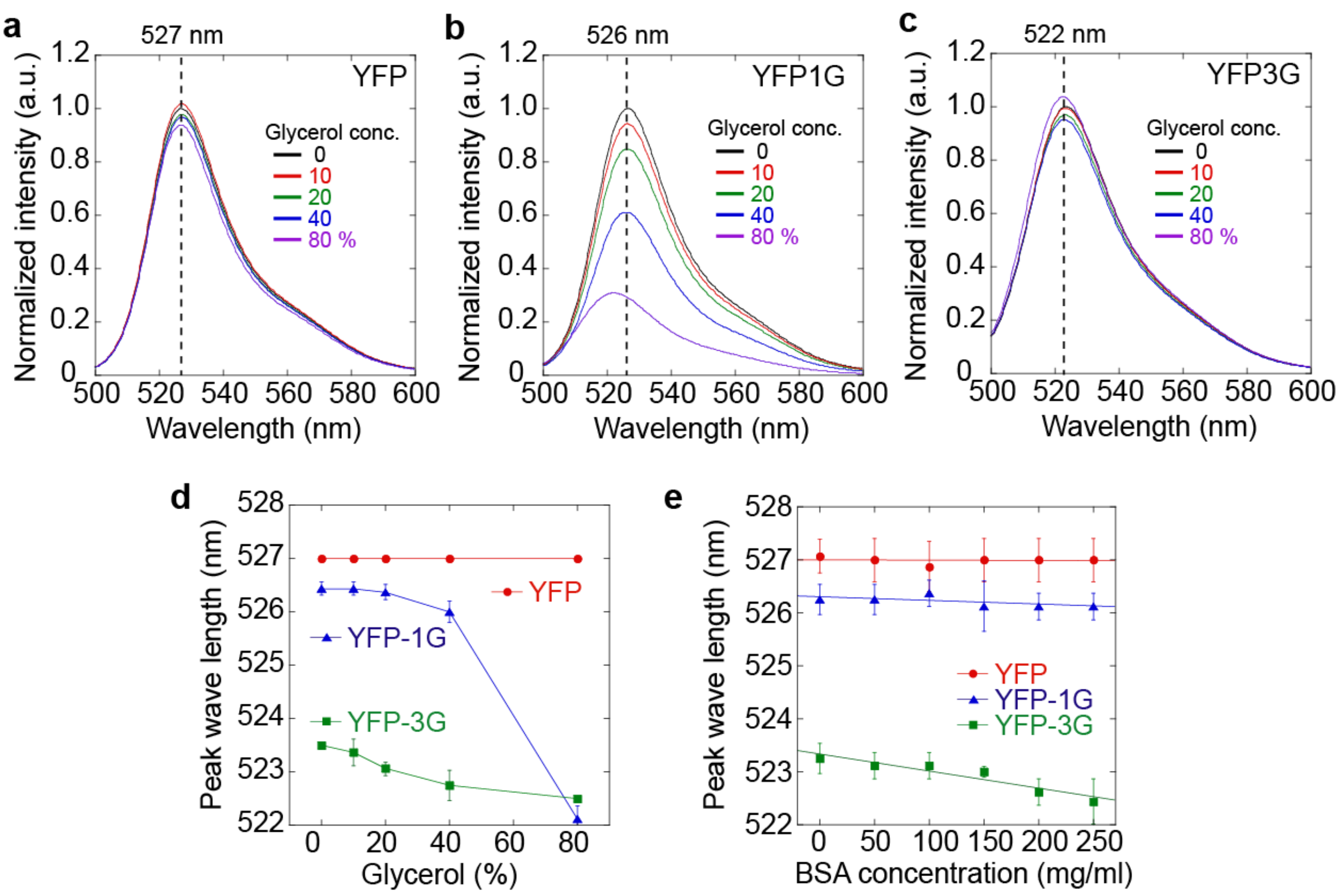

Figure S24. Dependences of spectra of YFP and glycine-inserted mutants on glycerol.

(**a,b,c**) Fluorescent spectra of YFP (**a**), YFP1G (**b**), and YFP3G (**c**), at 0–80% Glycerol (*black*, 0%; *red*, 10%; *green*, 20%; *blue*, 40%; *purple*, 80%). Excitation wavelengths were 488 nm. Traces represent the average of four individual trials. (**d,e**) Glycerol dependence (**d**) and BSA dependence (**e**) of the peak fluorescence intensities of YFP (red), YFP-1G (blue), and YFP-3G (green). The values are normalized with the value at 0 mg/ml BSA. Error bars, standard deviation.

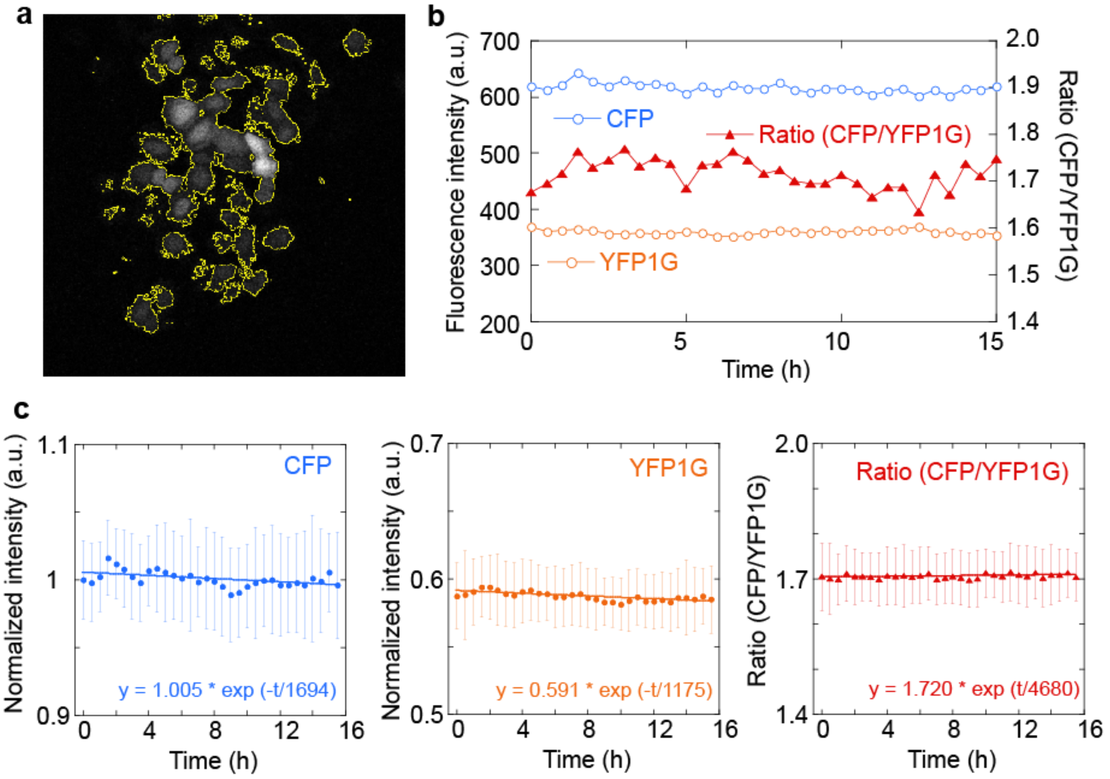

Figure S25. Photobleaching of GimRET fluorescence.

(**a**) Superimposed fluorescent images and cell boundaries detected by imaging processing (*yellow lines*). The original image was blurred by a Gaussian filter, and a histogram of the fluorescence intensity of each pixel was constructed from the filtered image. The threshold between cells and the other area was defined as threefold the peak value of the background in the histogram. (**b**) Mean fluorescent intensity of the CFP (*cyan*) and YFP1G channels (*orange*) and the ratio (CFP/YFP1G) (*red*). Each plot is the averaged fluorescent intensity of pixels inside the cell boundary (**a**, *yellow line*). (**c**) Averaged mean fluorescent intensity of the CFP (*left*) and YFP1G channels (*middle*) and the ratio (*right*) of 16 trials. The solid lines fits to a single exponential decay curve, $f(t) = a \cdot \exp(-t/\tau)$ or $a \cdot \exp(t/\tau)$. Error bars, standard deviations. Because photobleaching of the YFP1G channel originated from both photobleaching of YFP and CFP, the decay time of the YFP1G fluorescence was smaller than that of CFP. Differences between the decay times of CFP and YFP1G increased the intensity ratio. In our experimental setup, the time constant of the intensity ratio increase was 4680 h, which is negligibly longer than the observation period.

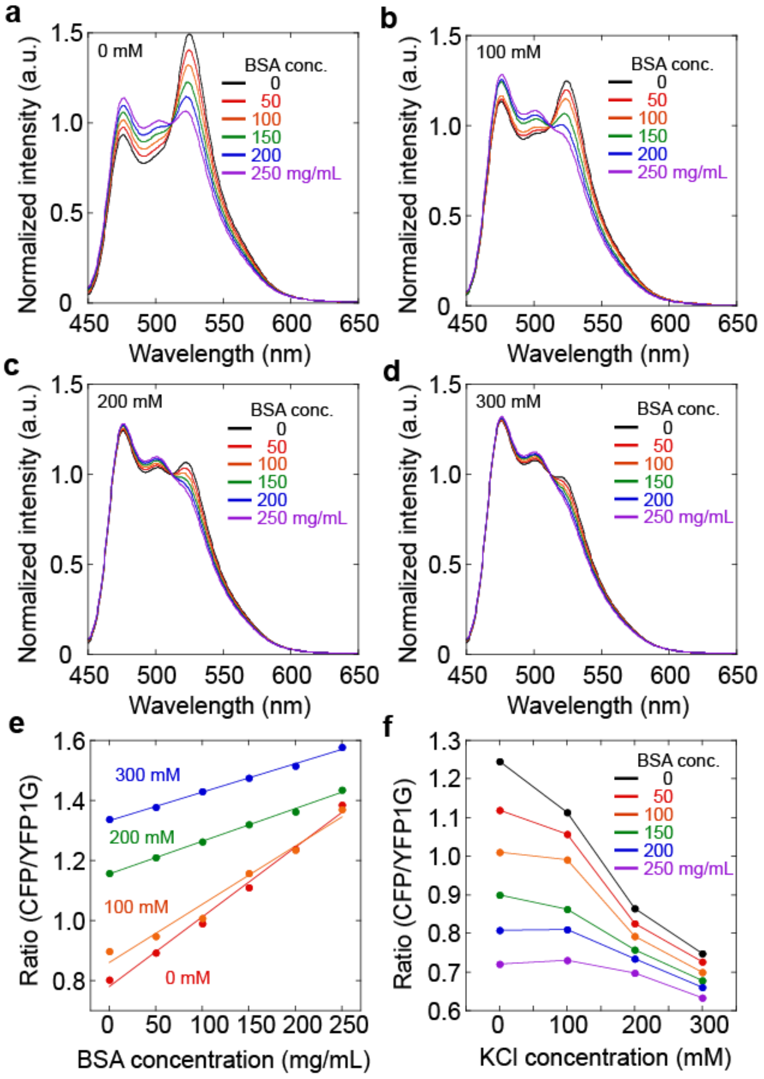

Figure S26. BSA concentration dependence of GimRET at various Cl$^-$ concentrations.

(**a–d**) Fluorescence spectra of GimRET at 0–250 mg/mL BSA (*black*, 0 mg/mL; *red*, 50 mg/mL; *orange*, 100 mg/mL; *green*, 150 mg/mL; *blue*, 200 mg/mL; *purple*, 250 mg/mL) containing 0 mM (**a**), 100 mM (**b**), 200 mM (**c**), or 300 mM (**d**) KCl. The excitation wavelength was 440 nm. Traces represent the average of four individual trials. (**e**) Relationship between BSA concentration and the ratio of the intensity between 460–500 nm and 520–560 nm at 0 mM (*red*), 100 mM (*orange*), 200 mM (*green*), and 300 mM (*blue*) KCl concentration. (**f**) Relationship between the ratio of intensity and the KCl concentration at 0–250 mg/mL BSA (*black*, 0 mg/mL; *red*, 50 mg/mL; *orange*, 100 mg/mL; *green*, 150 mg/mL; *blue*, 200 mg/mL; *purple*, 250 mg/mL). Traces or plots represent the average of four individual trials.

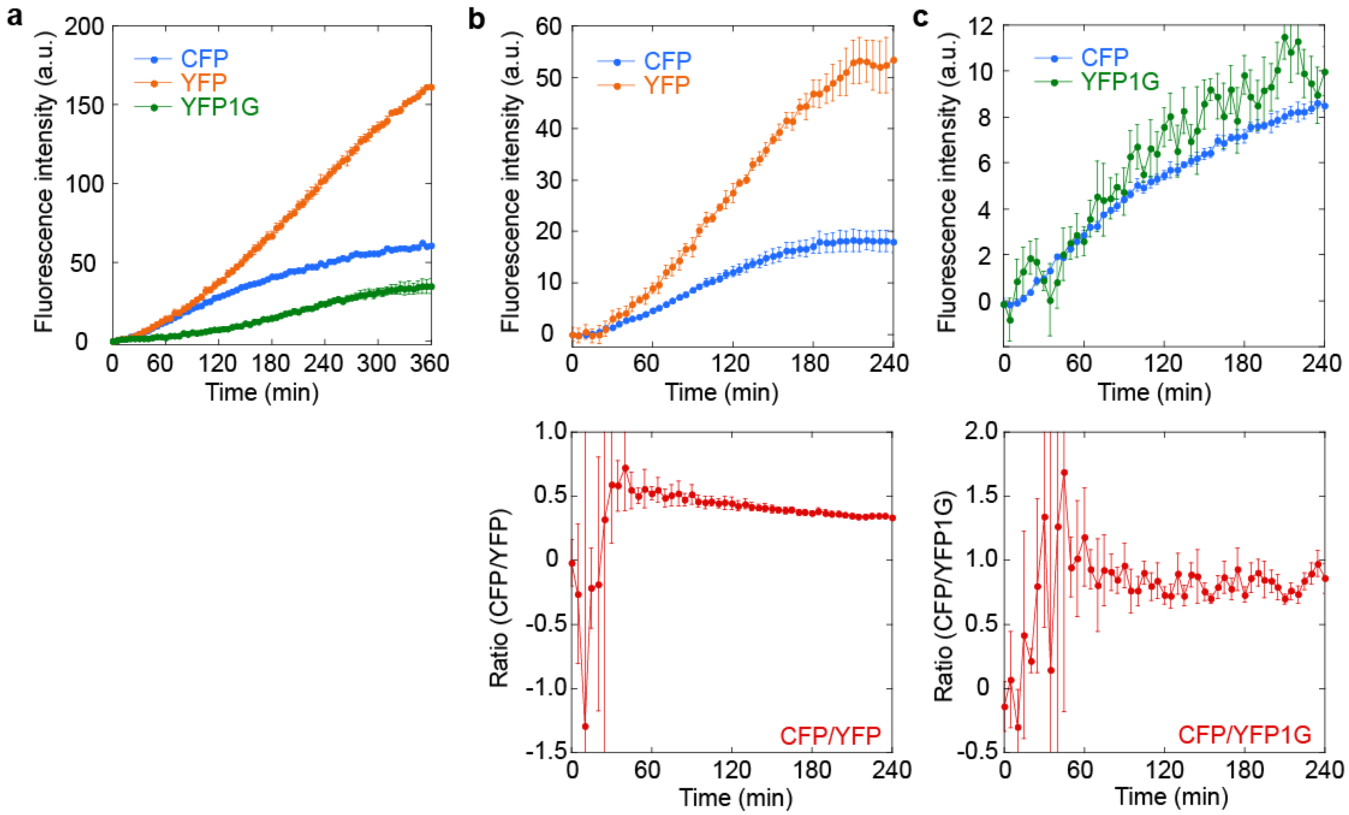

Figure S27. Folding and maturation of CFP, YFP, and YFP1G in *E. coli*.

(**a**) Average traces of the fluorescence intensity of *E. coli* during expression of CFP (*blue*), YFP (*orange*), and YFP1G (*green*). (**b**) Average traces of the fluorescence intensity of *E. coli* expressing CFP (*blue*) and YFP (*orange*) in CFP-YFP and the intensity ratio (*red*). (**c**) Average traces of the fluorescence intensity of *E. coli* expressing CFP (*blue*) and YFP1G (*green*) in GimRET and the intensity ratio (*red*). All traces represent the average of four individual trials. Error bars, standard deviations.

The procedure of the Measurement of folding and maturation of fluorescent protein in *E. coli* was performed following the previous report (7). *E. coli* transformed by the plasmid of each fluorescent protein were cultured for 12 h in LB medium. *E. coli* was collected by centrifuging and dissolved in M9 medium. From just after the induction by 1 μM IPTG, the fluorescent intensity of the *E. coli* were measured using Multilabel Reader 2030 ARVO X3 (Perkin Elmer, MA) at 37°C for 6 h. The excitation wavelength was set to 430 nm for CFP, and 510 nm for YFP1G and YFP. The emission wavelength was set to 475–485 nm for CFP, and 535–545 nm for YFP1G and YFP.

7 Lee, S. K., & Keasling, J. D. *Appl Environ Microbiol.* **71**, 6856-6862 (2005).

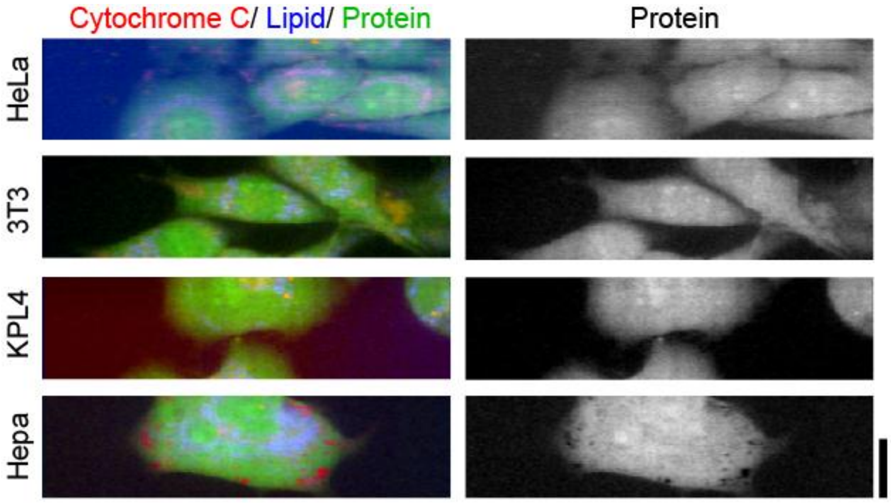

Figure S28. Raman images of four cell-lines.

(*Left panels*) RGB reconstituted Raman images of HeLa, 3T3, KPL4, and Hepa1-6. Raman peaks at 753 cm^{-1} (cytochrome C), 1686 cm^{-1} (proteins), and 2852 cm^{-1} (lipids) are mapped in blue, green, and red, respectively. (*Right panels*) Grey scale images of HeLa, 3T3, KPL4, and Hepa1-6 in the peak intensity of 1686 cm^{-1} (proteins). Scale bar, 10 µm.

Cells were plated on silica coverslip (SPI supplies, West Chester, PA) coated with 0.1% gelatine and cultured for 3 days. The medium was replaced with Tyrode's solution just before the observation to remove the background Raman signals from the components in the medium. We used a home-built line-scanning Raman microscope with a 60× water immersion lens with 1.27 numerical aperture (NIKON CFI Plan Apo IR) and a laser for 532 nm excitation wavelength (8). The exposure time of each line was 5 s, and the laser intensity was 2.4 mW/μm^2.

8 Ichimura, T. et al., *PLoS One* **9**, e84478 (2014).

Supplemental Video

Movie S1. Time-lapse movie of HeLa cells expressing GimRET.

The images were taken with a multi-photon microscope (FV1000-MPE, Olympus, JP) with a 20× objective lens (NA 1.05, water, XLPLN, Olympus, JP) every 20 min for 25 hours. The size of images was 204.2×204.2 μm^2 (512×512 pixels), and the pixel dwell time was 0.497 µs. The colour (Black–Blue–Green–Yellow-Red-While) indicates the GimRET ratio from 1.0 to 3.0. One sec in the movie corresponds to 5 hours in real time.
